# Using hyperpolarised NMR and DFT to rationalise the unexpected hydrogenation of quinazoline to 3,4-dihydroquinazoline†
†Raw NMR and DFT data can be found at: https://doi.org/10.15124/9294945a-8805-41bb-a3a5-ea77e36ec1a0
‡
‡Electronic supplementary information (ESI) available: NMR and DFT data. CCDC 1556095. For ESI and crystallographic data in CIF or other electronic format see DOI: 10.1039/c8cc04826f


**DOI:** 10.1039/c8cc04826f

**Published:** 2018-08-28

**Authors:** Josh E. Richards, Alexander J. J. Hooper, Oliver W. Bayfield, Martin C. R. Cockett, Gordon J. Dear, A. Jonathon Holmes, Richard O. John, Ryan E. Mewis, Natalie Pridmore, Andy D. Roberts, Adrian C. Whitwood, Simon B. Duckett

**Affiliations:** a Centre for Hyperpolarisation in Magnetic Resonance , University of York , Heslington , York YO10 5NY , UK . Email: simon.duckett@york.ac.uk; b GlaxoSmithKline Research and Development Ltd , Park Road , Ware , Hertfordshire SG12 0DP , UK

## Abstract

PHIP and SABRE hyperpolarized NMR methods are used to follow the unexpected metal-catalysed hydrogenation of quinazoline (Qu) to 3,4-dihydroquinazoline as the sole product. A solution of [IrCl(IMes)(COD)] in dichloromethane reacts with H_2_ and Qu to form [IrCl(H)_2_(IMes)(Qu)_2_] (**2**). The addition of methanol then results in its conversion to [Ir(H)_2_(IMes)(Qu)_3_]Cl (**3**) which catalyses the hydrogenation reaction. Density functional theory calculations are used to rationalise a proposed outer sphere mechanism in which (**3**) converts to [IrCl(H)_2_(H_2_)(IMes)(Qu)_2_]Cl (**4**) and neutral [Ir(H)_3_(IMes)(Qu)_2_] (**6**), both of which are involved in the formation of 3,4-dihydroquinazoline *via* the stepwise transfer of H^+^ and H^–^, with H_2_ identified as the reductant. Successive ligand exchange in **3** results in the production of thermodynamically stable [Ir(H)_2_(IMes)(3,4-dihydroquinazoline)_3_]Cl (**5**).

## Abstract 2

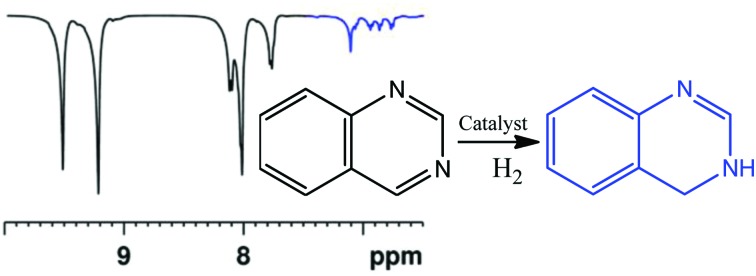
Hyperpolarised NMR allows the tracking of the DFT-rationalised outer sphere hydrogenation of quinazoline.

## 

The development and refinement of hyperpolarization methods over the past 20 years has enhanced the power of nuclear magnetic resonance (NMR) as a spectroscopic tool while leading to clinical magnetic resonance imaging (MRI) applications.^1^–^4^ The most well-established of these techniques, dynamic nuclear polarization (DNP), has been used in areas as diverse as materials characterisation,^5^,^6^ mechanistic and kinetic studies of chemical and enzymatic transformations by stopped flow DNP-NMR,^7^–^9^ and *in vivo*^1^H, ^13^C and ^15^N monitoring.^10^–^12^ Other approaches have exploited *para*-hydrogen (*p*-H_2_) as an agent to transfer polarization. *para*-Hydrogen Induced Polarization (PHIP)^13^ was pioneered by Weitekamp,^14^,^15^ Bargon^16^ and Eisenberg,^13^,^17^ and normally requires an unsaturated molecule to act as a hydrogen acceptor. Reaction products formed by integrating protons from the *p*-H_2_ are created with non-equilibrium nuclear spin distributions and as a result yield greatly enhanced NMR signals.^18^ This approach has been used widely to investigate the reactivity of metal hydride complexes and probe their role in, for example, metal catalysed hydrogenation^19^–^21^ and hydroformylation^22^–^24^ reactions. Other applications of PHIP have seen *p*-H_2_ used to sensitize MRI studies,^25^–^28^ to probe heterogeneous reactions^29^–^31^ and metabolism^32^ and more recently to create long-lived magnetic states.^33^–^35^

The requirement for chemical modification in PHIP has been addressed through the Signal Amplification By Reversible Exchange (SABRE) hyperpolarization method which, rather than relying on the hydrogenation of an unsaturated substrate, instead uses a metal complex as a chemical intermediary to bring the sample into temporary contact with *p*-H_2_.^36^–^38^ This method has been shown to polarize a wide range of substrates, leading to very large MR signal enhancements^39^ in the liberated substrate. Theoretical treatments have rationalized this process in terms of the *J*-coupling interactions that exist in these labile complexes^40^,^41^ whilst subsequent developments have led to the production of hyperpolarised long-lived singlet states using SABRE.^42^,^43^

In this paper both PHIP and SABRE are used to follow the unexpected metal-catalysed hydrogenation of quinazoline (Qu) to 3,4-dihydroquinazoline. Density Functional Theory (DFT) is combined with the experimental observations to rationalise a proposed outer sphere mechanism for the reaction. The experimental process starts with the reaction of a dichloromethane-d_2_ solution of [IrCl(COD)(IMes)], **1**,^44^ (IMes = 1,3-bis(2,4,6-trimethylphenyl)imidazole-2-ylidene and COD = cyclooctadiene) with Qu and H_2_. Rather than yielding the Qu analogue of [Ir(H)_2_(IMes)(Qu)_3_]Cl which forms from the analogous reaction with quinoline,^45^ neutral [IrCl(H)_2_(IMes)(Qu)_2_] (**2**) forms according to Scheme 1. The ^1^H NMR spectrum of **2** at 298 K yields two inequivalent hydride ligand signals at *δ* –22.84 (H_a_, linewidth 16.7 Hz) and *δ* –23.79 (H_b_, linewidth 16.3 Hz). When *p*-H_2_ is employed as the reactant at 253 K these two hydride resonances exhibit weak PHIP enhancement^37^ which confirms their assignment as a pair of *cis* hydride ligands. In addition, the proton resonances attributable to the Qu ligand *trans* to the hydrides as well as those of free Qu show weak SABRE enhancement thus confirming the transient binding of the Qu ligand to the metal centre.

**Scheme 1:**
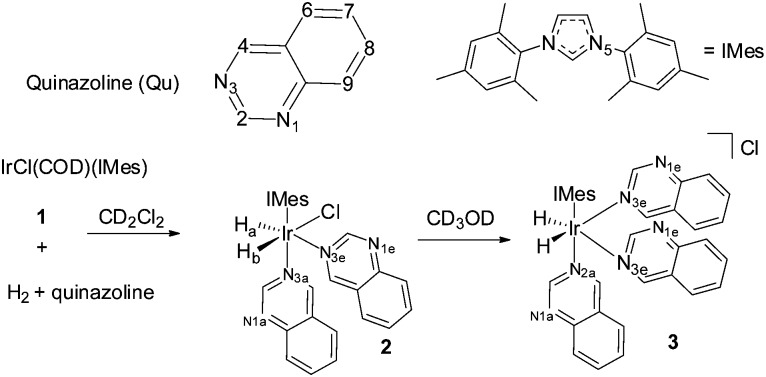
Solvent dependent reaction of **1** with quinazoline and H_2_ leads to **2** in dichloromethane and unstable **3** in methanol-d_4_.

When this process is repeated at 298 K, the two hydride ligands of **2** no longer exhibit PHIP, but all six of the free Qu aromatic proton resonances exhibit SABRE, with the degree of ^1^H signal enhancement ranging from 61-fold for H_8_ to 108-fold for H_2_. An average enhancement of 85-fold over all six protons of Qu was achieved for a concentration of **1** of 5 mM with a 17-fold ligand excess (Fig. 1). Full characterisation and details confirming the assignment of **2** to [IrCl(H)_2_(IMes)(Qu)_2_] are given the ESI.‡

**Fig. 1:**
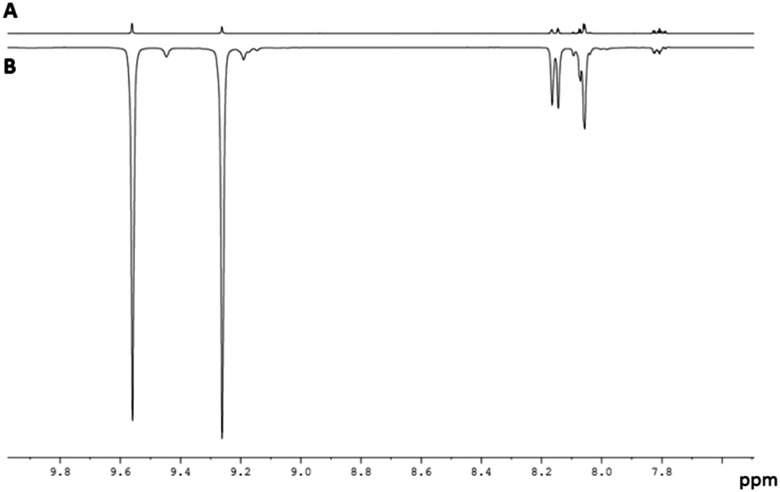
Typical ^1^H NMR spectra of the aromatic region of a sample consisting of Qu (0.1 M) and **1** (5 mM) in dichloromethane-d_2_: (A) magnetisation at thermal equilibrium and (B) hyperpolarised magnetisation created by SABRE at 65 G.

The fluxional behaviour of **2** in dichloromethane-d_2_ was then probed by EXSY methods over the temperature range 263 to 299 K. Its inequivalent hydrides, H_a_ and H_b_, were found to interchange positions, with H_2_ elimination and dissociative Qu loss also being detected. The H_2_ loss pathway shows a [H_2_] dependence which is consistent with the involvement of the intermediate [IrCl(H)_2_(η^2^-H_2_)(IMes)(Qu)] (**4**), a product that forms when the ligand-dissociation-product [IrCl(H)_2_(IMes)(Qu)] is trapped by H_2_ rather than Qu. This mechanism is consistent with the reported H_2_ exchange pathway of IrCl(H)_2_(η^2^-H_2_)(PPr_3_^i^)_2_.^47^,^48^ The associated rate data from these studies yielded values for Δ*H*^‡^ and Δ*S*^‡^ of 87.8 ± 1 kJ mol^–1^ and 75 ± 3 J K^–1^ mol^–1^, respectively, for Qu_equatorial_ loss in accordance with a dissociative first step and a relatively high Ir–N bond energy.^46^

DFT calculations were performed to corroborate the assignment of **2**. With dichloromethane solvation included at the PCM continuum level, the calculations showed dispersion-corrected **2** to be 38.5 kJ mol^–1^ more stable than [Ir(H)_2_(IMes)(Qu)_3_]Cl (**3**) at 298 K, consistent with the observation of **2** as the only reaction product in dichloromethane. With methanol solvation however, **2** is predicted to be just 8.8 kJ mol^–1^ more stable than **3** in agreement with the expectation that charge-stabilizing methanol increases the likelihood of formation of **3**.

Prompted by this result, a further experiment was conducted in which a dichloromethane-d_2_ solution of **1** containing a 20-fold excess of Qu and 50 μl of methanol-d_4_ was placed under *p*-H_2_. As anticipated, ^1^H NMR signals due to **3** now dominate with the six free Qu resonances showing substantial SABRE signal gains. However, over the course of the next few hours, the SABRE-enhanced NMR spectra showed dramatic changes that signalled the exclusive conversion of Qu into 3,4-dihydroquinazoline,^49^–^51^ with no evidence for the formation of the corresponding 1,2-dihydroquinazoline^52^ isomer (see ESI,‡ for further details of the characterisation).

When the reaction was repeated in neat methanol-d_4_ (Fig. 2), the SABRE ^1^H-NMR spectra initially show dominant hyperpolarized signals for Qu but with minor signals due to 3,4-dihydroquinazoline present from the start. This suggests that the conversion of Qu to 3,4-dihydroquinazoline proceeds rapidly under these conditions. While all seven ring protons of 3,4-dihydroquinazoline show SABRE, no visible signal enhancement is seen for its exchangeable NH proton. In addition to these changes in the aromatic region, a single hydride signal emerges from a complex array of peaks at *δ* –23.6. This signal is associated with the 3,4-dihydroquinazoline analogue of **3**, [Ir(H)_2_(IMes)(3,4-dihydroquinazoline)_3_]Cl (**5**), as characterised by NMR and whose relatively greater stability than **3** was validated by DFT (3 kJ mol^–1^). This complex hydride resonance behaviour reflects the formation of metal complex products that feature varying proportions of 3,4-dihydroquinazoline and quinazoline ligands with **5** the most stable of these and ultimately dominating the spectrum. At the completion of the reaction, **5** delivers hyperpolarised signals for 3,4-dihydroquinazoline at *δ* 7.1, 6.96, 6.88, 6.68 and 4.56 that exhibit enhancement factors of 96-, 40-, 40-, 35- and 26-fold respectively. Their average enhancements are lower than those of quinazoline which is consistent with the DFT prediction that **5** is more stable than **3**.

**Fig. 2:**
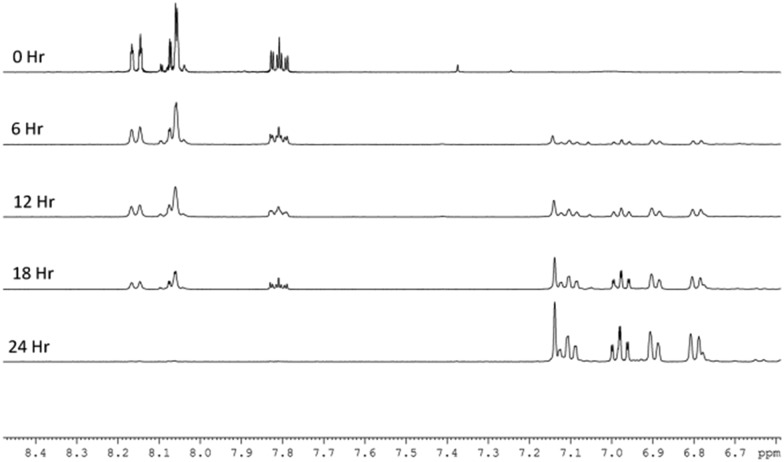
Plot of a series of expansions of the aromatic region of five ^1^H NMR spectra that track the conversion of quinazoline into 3,4-dihydroquinazoline in methanol over a 24 h period.

A series of control reactions were then used to probe this process in more detail. In the first, the original dichloromethane-d_2_ solution of **2** and Qu was heated under a H_2_ atmosphere overnight at 323 K in the absence of methanol. No evidence for the hydrogenation of Qu was observed and hence **2** cannot be involved in this process. A direct inner sphere mechanism involving the stepwise insertion of quinazoline into the Ir-H bonds of **3** might be predicted but our DFT calculations suggest that this process has an inhibiting energy barrier of >175 kJ mol^–1^. A second possibility involving transfer hydrogenation^53^–^56^ that works well with related substrates^57^ would involve methanol as the proton source.^58^ In order to identify the reductant, we placed a solution of **3** in pure methanol under H_2_ and observed gradual conversion of quinazoline to 3,4-dihydroquinazoline. Replacing the H_2_ atmosphere with N_2_ stopped the hydrogenation completely with no further increases observed in the 3,4-dihydroquinazoline ^1^H NMR signal over the following 24 h period. Subsequent addition of ammonium formate, a known proton source for transfer hydrogenation,^59^–^61^ failed to initiate any further hydrogenation, even at 323 K, and so, on this basis, we conclude that the reductant must be H_2_.

We note that hydrogenation of 2-methylquinoline has been reported to take place *via* an outer sphere mechanism involving an iridium dihydrogen dihydride complex.^62^,^63^ Such a mechanism is consistent with our SABRE results and the dispersion-corrected DFT calculations used here to underpin Scheme 2 (see ESI‡). Thus, we propose a mechanism by which **3** first forms [Ir(H)_2_(H_2_)(IMes)(Qu)_2_]Cl (**4**) (33 kJ mol^–1^ higher in energy than **3** as determined by the DFT calculations), before conversion to neutral [Ir(H)_3_(IMes)(Qu)_2_] (**6**) and protonated quinazoline (64.7 kJ mol^–1^ higher in energy) *via* reaction intermediate [Ir(H)_2_(IMes)(Qu)_2_···H···H···Qu]Cl. Hydride ligand transfer then follows to form 3,4-dihydroquinazoline (overall reaction exothermic by –33.7 kJ mol^–1^ at 298 K). As the catalytic cycle continues, successive ligand exchange in **3** results eventually in the thermodynamically stable metal complex product, **5**. The importance of the choice of methanol as solvent in realising the hydrogenation reaction lies in its role in stabilising **3** as a product of the reaction of **1** with Qu and H_2_ rather than acting as a proton source for the transfer hydrogenation step.

**Scheme 2:**
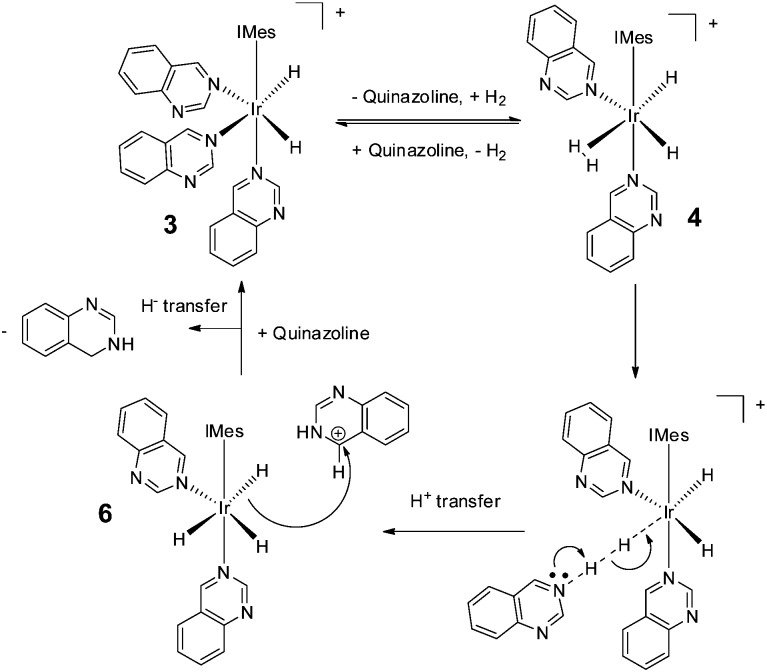
Route for the outer-sphere conversion of quinazoline into 3,4-dihydroquinazoline by **3** as determined by DFT.

In summary, we have shown that ^1^H NMR spectroscopy combined with SABRE can be used to follow the metal-catalysed hydrogenation of quinazoline exclusively to 3,4-dihydroquinazoline. While [IrCl(H)_2_(IMes)(Qu)_2_] (**2**) is unable to catalyse this transformation, the reaction proceeds readily *via* [IrCl(H)_2_(IMes)(Qu)_3_]Cl (**3**) with [Ir(H)_2_(IMes)(3,4-dihydroquinazoline)_3_]Cl (**5**) the thermodynamically favoured metal complex product. The reductant in this process is H_2_, and a solvent-dependent outer-sphere mechanism is proposed which requires the formation of [Ir(H)_2_(H_2_)(IMes)(Qu)_3_]Cl (**4**) and [Ir(H)_3_(IMes)(Qu)_2_] (**6**). Both **2** and **3** act as good SABRE catalysts with quinazoline as substrate whilst **5** performs the same function with 3,4-dihydroquinazoline. All three of these complexes, when monitored by NMR spectroscopy, show high levels of hyperpolarisation in the heteroatom containing rings.

We are grateful for support from the Wellcome Trust (grants 092506 and 098335), the EPSRC (EP/G009546/1 and EP/H029575/1) and GlaxoSmithKline (studentship, AJJH). Bruker BioSpin provided equipment and support through Dr J. A. B. Lohman, Dr D. Kilgour and colleagues.

## Conflicts of interest

There are no conflicts to declare.

## Supplementary Material

Supplementary information

Crystal structure data

## References

[cit1] Guenther U. L., Modern NMR Methodology, 2013, vol. 335, pp. 23–69.

[cit2] Keshari K. R., Wilson D. M. (2014). Chem. Soc. Rev..

[cit3] Oros A. M., Shah N. J. (2004). Phys. Med. Biol..

[cit4] Viale A., Reineri F., Santelia D., Cerutti E., Ellena S., Gobetto R., Aime S. (2009). Q. J. Nucl. Med. Mol. Imaging.

[cit5] Lee D., Monin G., Duong N. T., Lopez I. Z., Bardet M., Mareau V., Gonon L., De Paepe G. (2014). J. Am. Chem. Soc..

[cit6] Akbey U., Altin B., Linden A., Ozcelik S., Gradzielski M., Oschkinat H. (2013). Phys. Chem. Chem. Phys..

[cit7] Canet D., Lyon C. E., Scheek R. M., Robillard G. T., Dobson C. M., Hore P. J., van Nuland N. A. J. (2003). J. Mol. Biol..

[cit8] Chen H.-Y., Ragavan M., Hilty C. (2013). Angew. Chem., Int. Ed..

[cit9] Hilty C., Bowen S. (2010). Org. Biomol. Chem..

[cit10] Johanneson H., Macholl S., Ardenkjaer-Larsen J. H. (2009). J. Magn. Reson..

[cit11] Mayer D., Yen Y. F., Tropp J., Pfefferbaum A., Hurd R. E., Spielman D. M. (2009). Magn. Reson. Med..

[cit12] Ardenkjaer-Larsen J. H., Fridlund B., Gram A., Hansson G., Hansson L., Lerche M. H., Servin R., Thaning M., Golman K. (2003). Proc. Natl. Acad. Sci. U. S. A..

[cit13] Eisenschmid T. C., Kirss R. U., Deutsch P. P., Hommeltoft S. I., Eisenberg R., Bargon J., Lawler R. G., Balch A. L. (1987). J. Am. Chem. Soc..

[cit14] Pravica M. G., Weitekamp D. P. (1988). Chem. Phys. Lett..

[cit15] Bowers C. R., Weitekamp D. P. (1987). J. Am. Chem. Soc..

[cit16] Natterer J., Bargon J. (1997). Prog. Nucl. Magn. Reson. Spectrosc..

[cit17] Eisenberg R. (1991). Acc. Chem. Res..

[cit18] Anwar M. S., Blazina D., Carteret H. A., Duckett S. B., Halstead T. K., Jones J. A., Kozak C. M., Taylor R. J. K. (2004). Phys. Rev. Lett..

[cit19] Abdulhussain S., Breitzke H., Ratajczyk T., Gruenberg A., Srour M., Arnaut D., Weidler H., Kunz U., Kleebe H. J., Bommerich U., Bernarding J., Gutmann T., Buntkowsky G. (2014). Chem. Eng. J..

[cit20] Kovtunov K. V., Barskiy D. A., Shchepin R. V., Coffey A. M., Waddell K. W., Koptyug I. V., Chekmenev E. Y. (2014). Anal. Chem..

[cit21] Salnikov O. G., Kovtunov K. V., Barskiy D. A., Khudorozhkov A. K., Inozemtseva E. A., Prosvirin I. P., Bukhtiyarov V. I. and Koptyug I. V., Acs Catalysis, 2014, 4, 2022–2028.

[cit22] Fox D. J., Duckett S. B., Flaschenriem C., Brennessel W. W., Schneider J., Gunay A., Eisenberg R. (2006). Inorg. Chem..

[cit23] Godard C., Duckett S. B., Polas S., Tooze R., Whitwood A. C. (2005). J. Am. Chem. Soc..

[cit24] Godard C., Duckett S. B., Henry C., Polas S., Toose R., Whitwood A. C. (2004). Chem. Commun..

[cit25] Reineri F., Viale A., Giovenzana G., Santelia D., Dastru W., Gobetto R., Aime S. (2008). J. Am. Chem. Soc..

[cit26] Goldman M., Johannesson H., Axelsson O., Karlsson M. (2006). C. R. Chim.

[cit27] Hoevener J.-B., Schwaderlapp N., Borowiak R., Lickert T., Duckett S. B., Mewis R. E., Adams R. W., Burns M. J., Highton L. A. R., Green G. G. R., Olaru A., Hennig J., von Elverfeldtt D. (2014). Anal. Chem..

[cit28] Kovtunov K. V., Barskiy D. A., Coffey A. M., Truong M. L., Salnikov O. G., Khudorozhkov A. K., Inozemtseva E. A., Prosvirin I. P., Bukhtiyarov V. I., Waddell K. W., Chekmenev E. Y., Koptyug I. V. (2014). Chem. Eng. J..

[cit29] Wang W. Y., Xu J., Zhao Y. X., Qi G. D., Wang Q., Wang C., Li J. L., Deng F. (2017). Phys. Chem. Chem. Phys..

[cit30] Barskiy D. A., Kovtunov K. V., Gerasimov E. Y., Phipps M. A., Salnikov O. G., Coffey A. M., Kovtunova L. M., Prosvirin I. P., Bulditiyarov V. I., Koptyug I. V., Chekmenev E. Y. (2017). J. Phys. Chem. C.

[cit31] Kovtunov K. V., Barskiy D. A., Shchepin R. V., Salnikov O. G., Prosvirin I. P., Bukhtiyarov A. V., Kovtunova L. M., Bukhtiyarov V. I., Koptyug I. V., Chekmenev E. Y. (2016). Chem. Eng. J..

[cit32] Cavallari E., Carrera C., Aime S., Reineri F. (2017). Chem. Eng. J..

[cit33] Vinogradov E., Grant A. K. (2008). J. Magn. Reson..

[cit34] Canet D., Bouguet-Bonnet S., Aroulanda C., Reineri F. (2007). J. Am. Chem. Soc..

[cit35] Graafen D., Franzoni M. B., Schreiber L. M., Spiess H. W., Munnemann K. (2016). J. Magn. Reson..

[cit36] Adams R. W., Aguilar J. A., Atkinson K. D., Cowley M. J., Elliott P. I. P., Duckett S. B., Green G. G. R., Khazal I. G., Lopez-Serrano J., Williamson D. C. (2009). Science.

[cit37] Duckett S. B., Mewis R. E. (2012). Acc. Chem. Res..

[cit38] Green R. A., Adams R. W., Duckett S. B., Mewis R. E., Williamson D. C., Green G. G. R. (2012). Prog. Nucl. Magn. Reson. Spectrosc..

[cit39] Rayner P. J., Burns M. J., Olaru A. M., Norcott P., Fekete M., Green G. G. R., Highton L. A. R., Mewis R. E., Duckett S. B. (2017). Proc. Natl. Acad. Sci. U. S. A..

[cit40] Adams R. W., Duckett S. B., Green R. A., Williamson D. C., Green G. G. R. (2009). J. Chem. Phys..

[cit41] Ivanov K. L., Pravdivtsev A. N., Yurkovskaya A. V., Vieth H. M., Kaptein R. (2014). Prog. Nucl. Magn. Reson. Spectrosc..

[cit42] Roy S. S., Norcott P., Rayner P. J., Green G. G. R., Duckett S. B. (2016). Angew. Chem., Int. Ed..

[cit43] Theis T., Ortiz G. X., Logan A. W. J., Claytor K. E., Feng Y., Huhn W. P., Blum V., Malcolmson S. J., Chekmenev E. Y., Wang Q., Warren W. S. (2016). Sci. Adv..

[cit44] Torres O., Martin M., Sola E. (2009). Organometallics.

[cit45] Lloyd L. S., Adams R. W., Bernstein M., Coombes S., Duckett S. B., Green G. G. R., Lewis R. J., Mewis R. E., Sleigh C. J. (2012). J. Am. Chem. Soc..

[cit46] Pazderski L. (2008). Magn. Reson. Chem..

[cit47] Eckert J., Jensen C. M., Koetzle T. F., Lehusebo T., Nicol J., Wu P. (1995). J. Am. Chem. Soc..

[cit48] Li S. H., Hall M. B., Eckert J., Jensen C. M., Albinati A. (2000). J. Am. Chem. Soc..

[cit49] Burdick B. A., Benkovic P. A., Benkovic S. J. (1977). J. Am. Chem. Soc..

[cit50] Lewis J. C., Wiedemann S. H., Bergman R. G., Ellman J. A. (2004). Org. Lett..

[cit51] Makhloufi A., Wahl M., Frank W., Ganter C. (2013). Organometallics.

[cit52] Bugle R. C., Osteryoung R. A. (1979). J. Org. Chem..

[cit53] Gulcemal S., Gokce A. G., Cetinkaya B. (2013). Inorg. Chem..

[cit54] Modugno G., Monney A., Bonchio M., Albrecht M., Carraro M. (2014). Eur. J. Inorg. Chem..

[cit55] Zhu X.-H., Cai L.-H., Wang C.-X., Wang Y.-N., Guo X.-Q., Hou X.-F. (2014). J. Mol. Catal. A: Chem..

[cit56] Victoria Jimenez M., Fernandez-Tornos J., Perez-Torrente J. J., Modrego F. J., Winterle S., Cunchillos C., Lahoz F. J., Oro L. A. (2011). Organometallics.

[cit57] Rueping M., Antonchick A. P., Theissmann T. (2006). Angew. Chem., Int. Ed..

[cit58] Boutain M., Duckett S. B., Dunne J. P., Godard C., Hernandez J. M., Holmes A. J., Khazal I. G., Lopez-Serrano J. (2010). Dalton Trans..

[cit59] Drost R. M., Bouwens T., van Leest N. P., de Bruin B. and Elsevier C. J., Acs Catalysis, 2014, 4, 1349–1357.

[cit60] Smith D. D., Gallagher A. T., Crowley V. M., Gergens W. M., Abel P. W., Hulce M. (2014). Synthesis-Stuttgart.

[cit61] Talwar D., Salguero N. P., Robertson C. M., Xiao J. (2014). Chem. Eng. J..

[cit62] Dobereiner G. E., Nova A., Schley N. D., Hazari N., Miller S. J., Eisenstein O., Crabtree R. H. (2011). J. Am. Chem. Soc..

[cit63] Eisenstein O., Crabtree R. H. (2013). New J. Chem..

