# Supplementary material for: Using hyperpolarised NMR and DFT to rationalise the unexpected hydrogenation of quinazoline to 3,4-dihydroquinazoline
Source: Chem Commun (Camb). 2018 Aug 28;54(73):10375–8. doi: 10.1039/c8cc04826f (PMC6136267; doi:10.1039/c8cc04826f)
Supplement: Supplementary file 1 [file CC-054-C8CC04826F-s001.pdf]

Electronic Supplementary Material (ESI) for ChemComm.
This journal is © The Royal Society of Chemistry 2018

Using hyperpolarised NMR and DFT to rationalise the unexpected hydrogenation of quinazoline into 3,4-dihydroquinazoline

Josh E. Richards[a], Alexander J. J. Hooper[a], Oliver W. Bayfield[a], Martin C. R. Cockett[a], Gordon J. Dear[b], A. Jonathon Holmes[a], Richard O. John[a], Ryan E. Mewis[a], Natalie Pridmore[a], Andy D. Roberts[b] and Adrian C. Whitwood[a] and Simon B. Duckett[a]

[a]Department of Chemistry, University of York, Heslington, York, YO10 5DD, UK. YO10 5DD. Tel: +44 1904 432564; E-mail:simon.duckett@york.ac.uk

[b]GlaxoSmithKline Research and Development Ltd., Park Road, Ware, Hertfordshire, SG12 0DP, UK.

Supplementary Information

1. General considerations......3
3. Synthesis of 3,4-dihydroquinazoline......4
4. NMR Characterisation data for 2 collected in DCM......5
5. NMR characterisation for5 in MeOD......7
6. NMR characterisation data for 6 collected in d_4-MeOH......9
8. Hydride interconverison and exchange to free Hydrogen EXSY data for 2......11

1. General considerations

NMR Equipment. NMR measurements were made using a Bruker 400 MHz Avance III spectrometer equipped with either a TXO flow probe linked directly to the Polarizer[1] (for flow experiments) or BBI probe (for shake measurements). All measurements were made at 298 K.

*Para*hydrogen was prepared by flowing hydrogen gas over either charcoal at 30 K in a house-built apparatus (used in shake measurements) or iron oxide catalyst at 36 K (used in flow experiments).

Shake and drop' experiments: This method utilises a parahydrogen (p-H_2) source to fill the NMR tube which are then shaken in a specific stray magnetic field of the magnet. These areas were ~2G, and ~65G. It should be noted these are approximate values obtained from a gauss meter. All experiments were performed on the same 400 MHz NMR spectrometer unless stated otherwise. After each 'shake and drop' the sample is ejected, emptied of H_2 by vacuum and refilled with a fresh batch of p-H_2 (at 3 bar pressure). In temperature studies, the sample is left at a certain temperature (i.e. in ice or a heated water bath) for 3 minutes before shaking and inserting into the spectrometer.
To a Young's capped NMR tube was added the SABRE catalyst (~2 mg), the solvent and quinoxaline.

2. Synthesis of 3,4-dihydroquinazoline

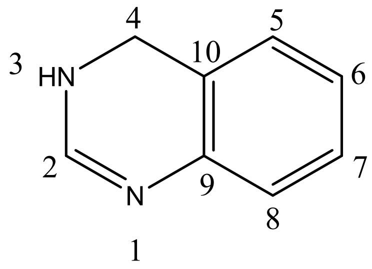

o-Aminobenzylamine (4.0 g, 33 mmol) was dissolved in anhydrous triethylorthoformate (20 ml, 120 mmol). The reaction was refluxed at 120°C for 14 hr and allowed to cool to room temperature. The reaction mixture was concentrated under reduced pressure to give a beige solid. The crude product was obtained and recrystallized from toluene to give a cream coloured solid (2.8 g, 21 mmol, 64.7 %). This was then stored in a glovebox.

Melting Point 123-126.5 (lit. 125-126)

Table S1: NMR data 3,4-dihydroquinazoline

Atom label	^{1}H resonance	^{13}C resonance	^{15}N resonance
1	10.45		135.0
2	7.17	147.1	
3			169.9
4	4.63	43.9 (J_{CH} 140.8, Ar$\underline{C}$HNH)	
5	6.90	126.0	
6	7.13	127.8 (J_{CH} 162.7, Ar$\underline{C}$)	
7	7.01	124.2 (J_{CH} 162.3, Ar$\underline{C}$)	
8	6.86	119.9 (J_{CH} 160.3, Ar$\underline{C}$)	
9		120.6	
10		139.9	

3. NMR Characterisation data for 2 collected in DCM

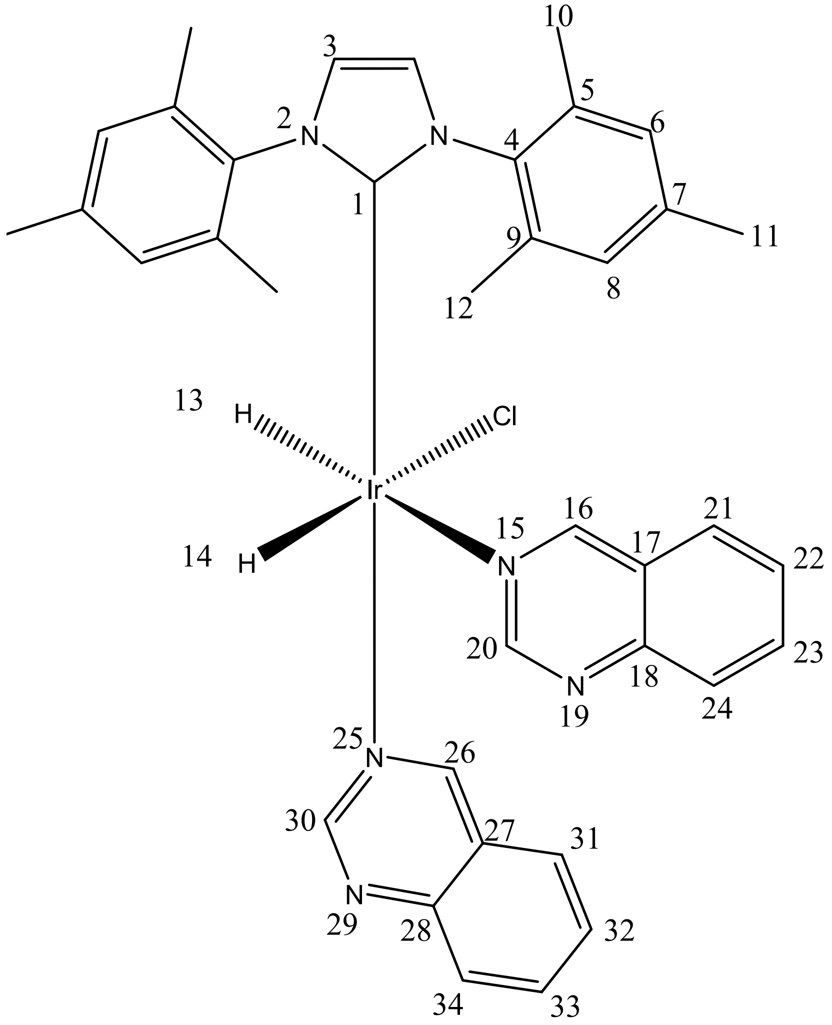

The formation of **2** was confirmed by a series of 1D NOE measurements that mapped interactions between the hydride ligands and the protons of the bound Qu ligands. Complete ligand assignment was achieved using COSY methods with heteronuclear multiple quantum correlation (HMQC) spectra being used to locate the corresponding ^{15}N signals associated with the bound Qu ligands. These spectra yielded ^{15}N chemical shift values for the N_1 site of free Qu at δ 281.3, where J_{NH} = 14.9 Hz, and the N_3 site at δ 291.8, with *ortho* J_{NH} values of 10.8 and 15 Hz (see Scheme 1 for site numbering). Five further ^{15}N resonances were located at δ 192.9, 226.2, 246.5, 282.9 and 283.6 in this spectrum. Of these, the signal at δ 192.9 arises from the IMes ligand's nitrogen centre while the bound N_{3e}-Qu site provides the signal at δ 246.5 which couples strongly (14 Hz) to hydride H_a. Thus their mutual *trans* orientation is confirmed. The N_{1e} signal of this ligand appears at δ 283.6 and connects to a δ 9.40 aromatic resonance through a J_{NH} coupling of 16 Hz. Hence upon binding to iridium, the ^{15}N shift of the Qu-N_{3e} moves to lower field by 45.3 ppm while that of the unbound N_1 site moves to higher field by 2.3 ppm. These shifts are of the same order as those observed for pyridine in the related complex *trans*-(H,N)(H,Cl)–[Ir(Py^{15}N)(P(C$_6$H$_5$)$_3$)$_2$.[46] The Ir-N_{3a} and N_{1a} resonances of the axial Qu ligand

of **2** appear at δ 226.2 and 282.9 respectively, with the N_{3a} binding shift of 65.6 ppm suggesting a stronger bonding interaction in the axial Qu ligand. Confirmation of the presence of the directly bound, but NMR silent, chloride ligand was obtained by the addition of $AgBF_4$ which resulted in conversion of **2** into the initially predicted *tris*–substituted cation $[Ir(H)_2(IMes)(Qu)_3]BF_4$.

Table S2: NMR data for **2** in DCM

Resonance number	^{1}H (ppm)	^{13}C (ppm)	^{15}N (ppm)
1		182.7	
2			192.9
3	6.83	121.6	
4		-	
5		-	
6	6.54	128.3	
7		-	
8	6.64	128.3	
9		-	
10	2.21	18.0	
11	1.79	20.5	
12	2.28	18.1	
13	−22.77, d, J=7.9 Hz		
14	−23.78, d, J=7.9 Hz		
15			246.5, J_{NH} = 14 Hz
16	10.08	159.1	
17		-	
18		-	
19			283.6, J_{NH} = 16 Hz
20	9.20	160.4	
21	7.94	128.2	
22	7.70	127.6	
23	7.79	127.4	
24	8.06	134.5	
25			226.2

26	9.73	160.8	
27		-	
28		-	
29			282.9
30	9.40	162.55	
31	7.66	127.4	
32	7.44	128.3	
33	7.97	128.2	
34	7.74	134.6	

4. NMR characterisation for 3 in MeOD

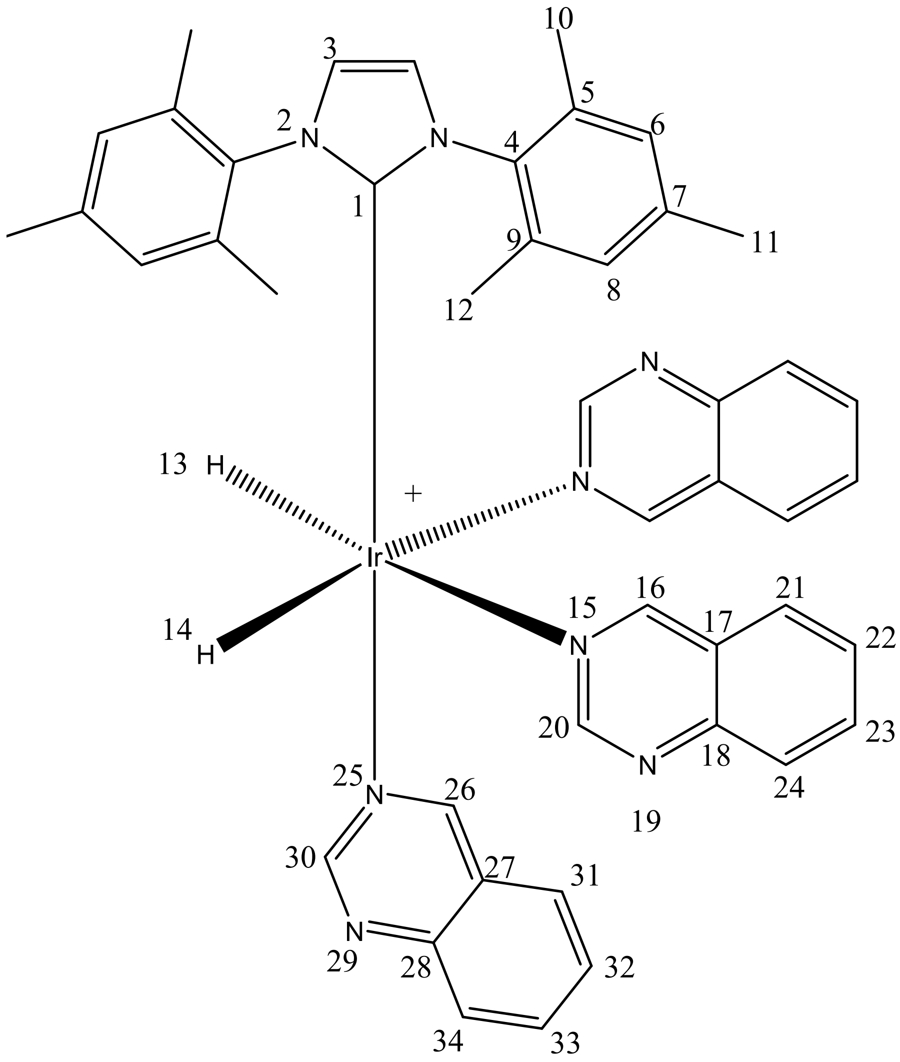

Table S3: NMR data for **3** in methanol

Resonance number	^{1}H (ppm)	^{13}C (ppm)	^{15}N (ppm)
1		179.59	
2			194.87
3	7.25	122.86	
4		136.76	
5		134.76	
6	6.27	128.17	
7		138.65	
8	6.33	128.77	
9		136.20	

10	2.21	19.89	
11	1.83	19.58	
12	2.12	17.78	
13	-21.87 @ 243 K, 7.42 Hz		
14	-21.87 @ 243 K, 7.42 Hz		
15			237.81, J_{NH} = 9 Hz
16	9.59	164.69, J_{CH} = 184.14	
17		147.40	
18		158.01	
19			282.50
20	9.16	158.05, J_{CH} = 207.63	
21	8.12	126.99	
22	7.84	129.09	
23	8.15	127.51	
24	8.07	128.06	
25			225.30
26	9.18	164.4, J_{CH} = 186.32	
27		147.44	
28		149.30	
29			285.10
30	9.30	160.74, J_{CH} = 208.68	
31	7.60	127.30	
32	7.97	135.90	
33	7.54	129.05	
34	7.89	127.37	

5. **NMR characterisation data for 5 collected in d_4-MeOH**

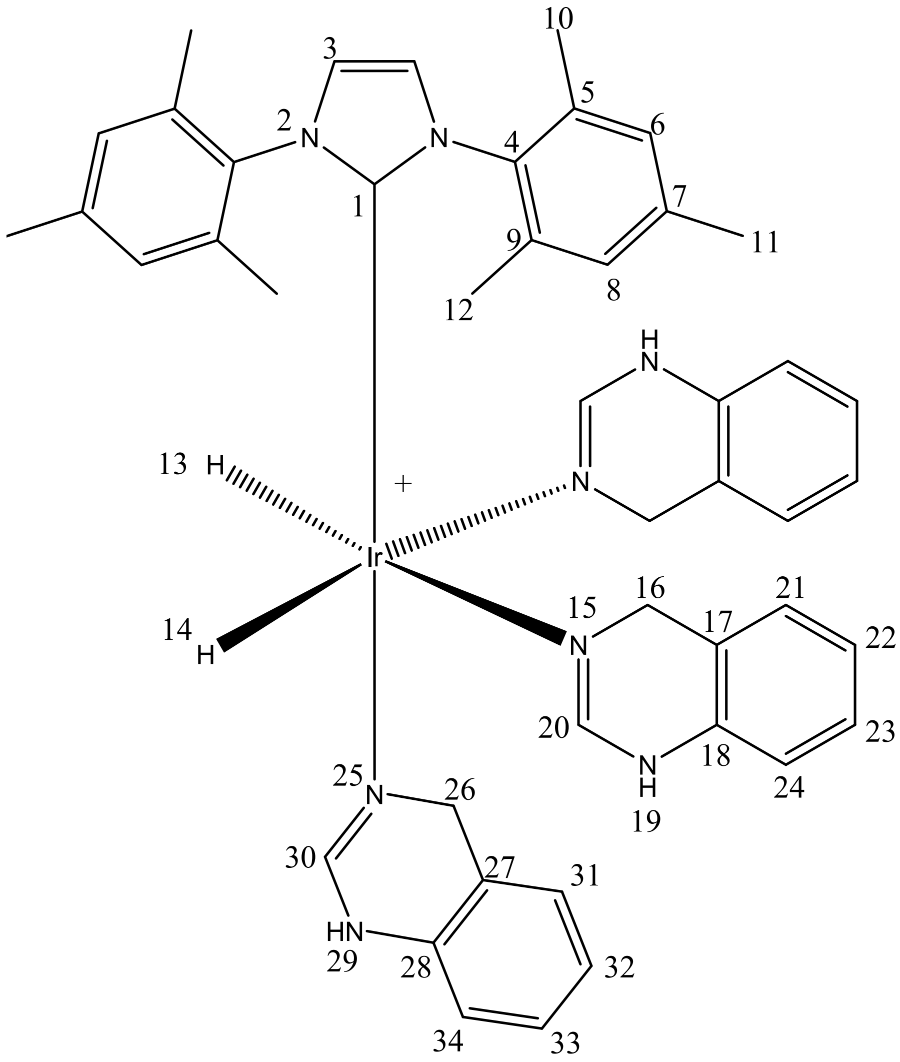

Table S4: NMR data for **5** in methanol

Resonance number	^{1}H (ppm)	^{13}C (ppm)	^{15}N (ppm)
1		160.2	
2			193.1
3	6.79	155.53	
4		138.4	
5		128.7	
6	6.78	120.5	
7		122.1	
8	6.78	120.5	
9		137.1	

10	2.19	20.4	
11	2.02	18.8	
12	2.19	20.4	
13	−23.5		
14	−23.5		
15			157.3
16	4.57	57.4	
17		138.2	
18		139.0	
19			--
20	6.98	154.3	
21	6.89	124.0	
22	6.80	125.8	
23	7.02	120.7	
24	6.64	119.9	
25			141.1
26	4.42	45.4	
27		143.1	
28			143
29			--
30	7.12	144.2	
31	6.47	117.5	
32	6.83	124.1	
33	6.99	120.8	
34	7.06	120.1	

6. Effect of time on inorganic product speciation

This change is reflected in the hydride region of the corresponding ^{1}H NMR spectra. After catalyst activation, just one hydride signal results due to **4** when the substrate is completely converted. As the complex is vastly outnumbered by the substrate, the hydride intensities are often very weak and only clearly visible through hyperpolarisation. As the reaction the proceeds the hydride region becomes complicated as 3,4-dihydroquinazoline competes with quinazoline for binding until eventually **5** dominates (Fig S1).

Figure S1. Series of four hyperpolarised ^{1}H NMR spectra showing the conversion of **4** (upper) into **5** (lower) with reaction time.

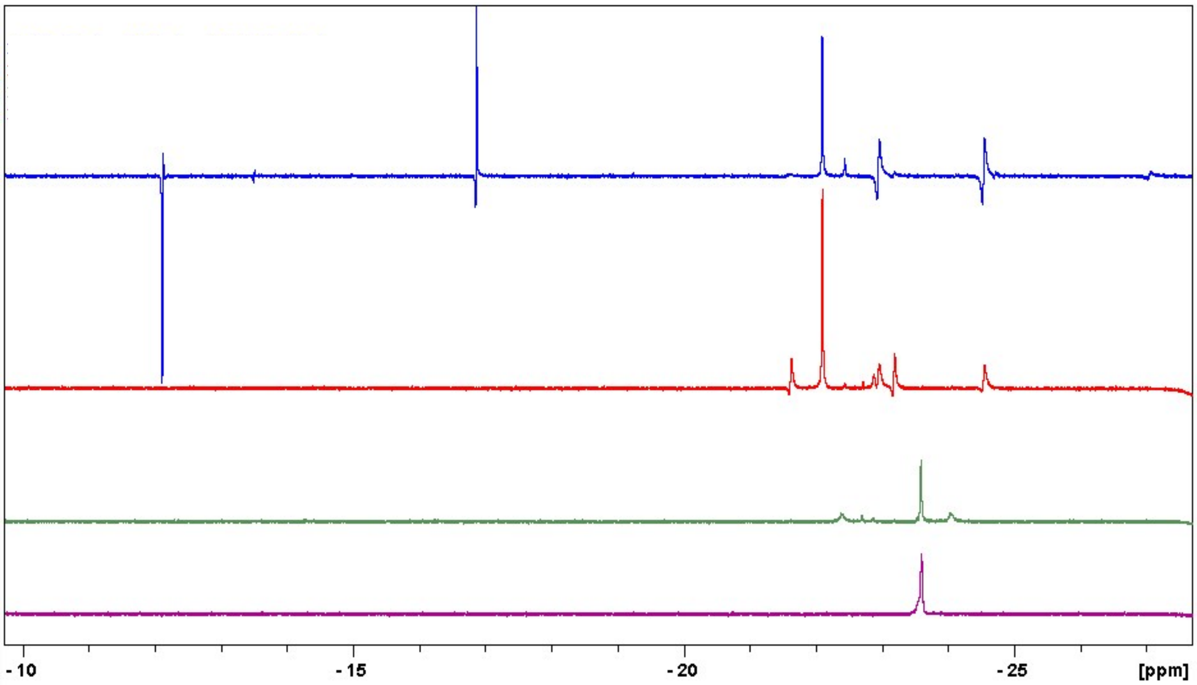

.

7. **Hydride ligand interconversion rate and rate of exchange into free hydrogen as determined via EXSY data for 2**

Table S5: EXSY data at 263 K

Relative integrals (o1p = -23.6)			Sum of integrals	Mixing time, d8 / s	Resonances as a % composition after mixing time		
**-23.0**	**-23.6**	**4.64 (H_2)**			**-22.9**	**-23.5**	**4.64 (H_2)**
0.00	1.00	0.09	1.09	0.60	0.00	91.41	8.59
0.00	1.00	0.12	1.12	0.70	0.00	89.18	10.82
0.00	1.00	0.13	1.13	0.80	0.00	88.37	11.63
0.00	1.00	0.14	1.14	0.90	0.00	87.70	12.30
0.00	1.00	0.16	1.16	1.00	0.00	86.57	13.43
0.00	1.00	0.20	1.20	1.10	0.00	83.49	16.51
**Relative integrals (o1p = -23.0)**			**Sum of integrals**	**Mixing time, d8 / s**	**Resonances as a % composition after mixing time**		
**-23.0**	**-23.6**	**4.64 (H_2)**			**-22.9**	**-23.5**	**4.64 (H_2)**
1.00	0.00	0.09	1.09	0.70	91.65	0.00	8.35
1.00	0.00	0.09	1.09	0.80	91.50	0.00	8.50
1.00	0.00	0.13	1.13	0.90	88.40	0.00	11.60
1.00	0.00	0.11	1.11	1.00	90.28	0.00	9.72
1.00	0.00	0.15	1.15	1.10	86.60	0.00	13.40
1.00	0.00	0.25	1.25	1.20	80.10	0.00	19.90

Table S6: EXSY data at 268 K

Relative integrals (o1p = -23.6)			Sum of integrals	Mixing time, d8 / s	Resonances as a % composition after mixing time		
**-23.0**	**-23.6**	**4.64 (H_2)**			**-22.9**	**-23.5**	**4.64 (H_2)**
0.04	1.00	0.13	1.17	0.30	3.78	85.35	10.87
0.05	1.00	0.14	1.20	0.40	4.45	83.49	12.06
0.06	1.00	0.18	1.24	0.50	4.80	80.76	14.44
0.11	1.00	0.25	1.36	0.60	8.07	73.53	18.40
0.15	1.00	0.40	1.55	0.70	9.83	64.38	25.79
0.28	1.00	0.55	1.82	0.80	15.21	54.84	29.95
0.34	1.00	0.77	2.10	0.90	15.96	47.56	36.48
0.39	1.00	1.00	2.38	1.00	16.18	41.99	41.83
**Relative integrals**			**Sum of**	**Mixing time,**	**Resonances as a % composition after mixing time**		

(o1p = -23.0)			integrals	d8 / s			
-23.0	-23.6	4.64 (H_2)			-22.9	-23.5	4.64 (H_2)
1.00	0.15	0.16	1.32	0.30	75.96	11.64	12.40
1.00	0.20	0.27	1.47	0.40	67.90	13.54	18.56
1.00	0.28	0.41	1.69	0.50	59.16	16.55	24.29
1.00	0.30	0.54	1.84	0.60	54.27	16.32	29.41
1.00	0.36	0.61	1.97	0.70	50.80	18.11	31.09
1.00	0.50	0.89	2.39	0.80	41.80	20.92	37.28
1.00	0.39	0.97	2.36	0.90	42.30	16.67	41.03
1.00	0.70	1.43	3.13	1.00	31.96	22.39	45.65

Table S7: EXSY data at 273 K

Relative integrals (o1p = -23.6)			Sum of integrals	Mixing time, d8 / s	Resonances as a % composition after mixing time		
-23.0	-23.6	4.64 (H_2)			-22.9	-23.5	4.64 (H_2)
0.05	1.00	0.05	1.10	0.10	4.45	91.04	4.51
0.12	1.00	0.12	1.24	0.20	9.36	80.93	9.72
0.21	1.00	0.22	1.42	0.30	14.56	70.20	15.24
0.25	1.00	0.32	1.58	0.40	16.15	63.45	20.40
0.30	1.00	0.41	1.72	0.50	17.66	58.28	24.06
0.42	1.00	0.59	2.01	0.60	20.97	49.68	29.35
0.43	1.00	0.76	2.20	0.70	19.77	45.53	34.70
0.50	1.00	1.01	2.51	0.80	19.98	39.91	40.11
0.51	1.00	1.20	2.71	0.90	18.91	36.88	44.21
0.53	1.00	1.32	2.84	1.00	18.58	35.16	46.26
**Relative integrals (o1p = -23.0)**			**Sum of integrals**	**Mixing time, d8 / s**	**Resonances as a % composition after mixing time**		
**-23.0**	**-23.6**	**4.64 (H_2)**			**-22.9**	**-23.5**	**4.64 (H_2)**
1.00	0.06	0.07	1.13	0.10	88.43	5.19	6.38
1.00	0.12	0.12	1.24	0.20	80.69	9.38	9.93
1.00	0.18	0.23	1.41	0.30	71.14	12.60	16.26
1.00	0.24	0.31	1.55	0.40	64.72	15.32	19.96
1.00	0.27	0.43	1.70	0.50	58.85	16.13	25.02
1.00	0.29	0.53	1.83	0.60	54.72	16.02	29.26
1.00	0.36	0.64	2.00	0.70	50.01	18.06	31.93
1.00	0.45	0.83	2.28	0.80	43.80	19.80	36.41
1.00	0.47	1.00	2.47	0.90	40.43	19.04	40.52

1.00	0.49	1.21	2.71	1.00	36.95	18.23	44.82

Table S8: EXSY data at 278 K

Relative integrals (o1p = -23.6)			Sum of integrals	Mixing time, d8 / s	Resonances as a % composition after mixing time		
-23.0	-23.6	4.64 (H_2)			-22.9	-23.5	4.64 (H_2)
0.12	1.00	0.11	1.23	0.10	9.76	81.61	8.63
0.27	1.00	0.24	1.51	0.20	17.65	66.15	16.20
0.41	1.00	0.45	1.87	0.30	22.16	53.59	24.25
0.48	1.00	0.69	2.16	0.40	22.06	46.24	31.70
0.63	1.00	1.00	2.63	0.50	23.87	38.04	38.09
0.69	1.00	1.17	2.86	0.60	24.07	34.92	41.01
0.66	1.00	1.34	3.00	0.70	22.06	33.30	44.64
0.90	1.00	2.15	4.05	0.80	22.23	24.69	53.08
0.93	1.00	2.54	4.47	0.90	20.83	22.37	56.80
**Relative integrals (o1p = -23.0)**			**Sum of integrals**	**Mixing time, d8 / s**	**Resonances as a % composition after mixing time**		
**-23.0**	**-23.6**	**4.64 (H_2)**			**-22.9**	**-23.5**	**4.64 (H_2)**
1.00	0.16	0.09	1.25	0.10	79.97	12.99	7.04
1.00	0.23	0.18	1.42	0.20	70.66	16.29	13.04
1.00	0.41	0.42	1.83	0.30	54.66	22.50	22.84
1.00	0.49	0.64	2.12	0.40	47.11	22.95	29.95
1.00	0.58	0.82	2.40	0.50	41.74	24.06	34.19
1.00	0.63	1.24	2.88	0.60	34.76	21.96	43.28
1.00	0.67	1.54	3.21	0.70	31.17	20.91	47.92
1.00	0.77	1.85	3.62	0.80	27.63	21.39	50.98
1.00	0.90	2.37	4.26	0.90	23.45	21.04	55.51

Table S9: EXSY data at 283 K

Relative integrals (o1p = -23.6)			Sum of integrals	Mixing time, d8 / s	Resonances as a % composition after mixing time		
-23.0	-23.6	4.64 (H_2)			-22.9	-23.5	4.64 (H_2)
0.15	1.00	0.10	1.25	0.05	11.81	80.02	8.17
0.29	1.00	0.23	1.52	0.10	19.29	65.58	15.12
0.42	1.00	0.39	1.81	0.15	23.23	55.25	21.52
0.49	1.00	0.62	2.11	0.20	23.41	47.36	29.23

0.62	1.00	0.88	2.50	0.25	24.85	39.93	35.22
0.66	1.00	1.06	2.71	0.30	24.23	36.85	38.92
0.79	1.00	1.32	3.11	0.35	25.45	32.12	42.43
0.86	1.00	1.71	3.58	0.45	24.14	27.94	47.92
0.89	1.00	2.26	4.15	0.55	21.55	24.08	54.37
**Relative integrals (o1p = -23.0)**			**Sum of integrals**	**Mixing time, d8 / s**	**Resonances as a % composition after mixing time**		
**-23.0**	**-23.6**	**4.64 (H_2)**			**-22.9**	**-23.5**	**4.64 (H_2)**
1.00	0.17	0.09	1.26	0.05	79.48	13.74	6.77
1.00	0.30	0.24	1.54	0.10	65.15	19.34	15.52
1.00	0.44	0.42	1.85	0.15	53.95	23.56	22.49
1.00	0.55	0.63	2.18	0.20	45.96	25.08	28.96
1.00	0.64	0.82	2.46	0.25	40.61	26.13	33.26
1.00	0.87	1.18	3.05	0.30	32.80	28.65	38.55
1.00	0.78	1.21	2.99	0.35	33.40	26.09	40.50
1.00	0.89	1.89	3.79	0.45	26.41	23.58	50.01
1.00	1.00	2.17	4.17	0.55	23.98	23.87	52.14

Table S10: EXSY data at 288 K

**Relative integrals (o1p = -23.6)**			**Sum of integrals**	**Mixing time, d8 / s**	**Resonances as a % composition after mixing time**		
**-23.0**	**-23.6**	**4.64 (H_2)**			**-22.9**	**-23.5**	**4.64 (H_2)**
0.07	1.00	0.06	1.12	0.01	6.12	88.90	4.98
0.16	1.00	0.12	1.29	0.03	12.56	77.74	9.70
0.30	1.00	0.24	1.54	0.05	19.70	64.85	15.45
0.55	1.00	0.64	2.19	0.10	25.06	45.59	29.34
0.61	1.00	1.05	2.66	0.15	23.05	37.59	39.36
0.70	1.00	1.48	3.18	0.20	22.03	31.49	46.48
0.74	1.00	1.91	3.65	0.25	20.32	27.37	52.31
0.82	1.00	2.45	4.27	0.30	19.24	23.44	57.32
0.81	1.00	2.56	4.38	0.35	18.54	22.86	58.60
0.85	1.00	3.16	5.01	0.40	17.03	19.95	63.02
0.87	1.00	3.46	5.34	0.50	16.34	18.74	64.92
**Relative integrals (o1p = -23.0)**			**Sum of integrals**	**Mixing time, d8 / s**	**Resonances as a % composition after mixing time**		
**-23.0**	**-23.6**	**4.64 (H_2)**			**-22.9**	**-23.5**	**4.64 (H_2)**
1.00	0.08	0.04	1.12	0.01	88.91	7.53	3.56

1.00	0.34	0.27	1.60	0.05	62.37	21.04	16.59
1.00	0.60	0.63	2.23	0.10	44.80	27.05	28.15
1.00	0.75	1.05	2.80	0.15	35.72	26.71	37.57
1.00	0.81	1.57	3.38	0.20	29.61	23.89	46.50
1.00	0.90	2.03	3.92	0.25	25.49	22.83	51.68
1.00	0.95	2.57	4.52	0.30	22.11	20.96	56.93
1.00	0.98	2.69	4.67	0.35	21.41	20.99	57.60

Table S11: EXSY data at 293 K

Relative integrals (o1p = -23.6)			Sum of integrals	Mixing time, d8 / s	Resonances as a % composition after mixing time		
**-23.0**	**-23.6**	**4.64 (H_2)**			**-22.9**	**-23.5**	**4.64 (H_2)**
0.04	1.00	0.03	1.06	0.01	3.61	93.93	2.46
0.13	1.00	0.12	1.25	0.02	10.48	80.02	9.50
0.17	1.00	0.16	1.34	0.02	12.89	74.88	12.23
0.26	1.00	0.25	1.51	0.03	17.50	66.08	16.42
0.27	1.00	0.28	1.55	0.03	17.69	64.53	17.78
0.30	1.00	0.34	1.64	0.04	18.37	60.86	20.78
0.36	1.00	0.41	1.77	0.04	20.24	56.45	23.31
0.39	1.00	0.53	1.92	0.05	20.23	52.08	27.70
0.47	1.00	0.73	2.21	0.06	21.46	45.29	33.25
0.55	1.00	0.98	2.52	0.08	21.69	39.61	38.70
0.55	1.00	1.23	2.78	0.10	19.72	36.00	44.28
**Relative integrals (o1p = -23.0)**			**Sum of integrals**	**Mixing time, d8 / s**	**Resonances as a % composition after mixing time**		
**-23.0**	**-23.6**	**4.64 (H_2)**			**-22.9**	**-23.5**	**4.64 (H_2)**
1.00	0.12	0.07	1.18	0.01	84.40	9.75	5.86
1.00	0.18	0.14	1.32	0.02	75.96	13.68	10.35
1.00	0.22	0.19	1.41	0.02	70.69	15.61	13.71
1.00	0.25	0.25	1.50	0.03	66.56	16.94	16.50
1.00	0.32	0.30	1.62	0.03	61.72	19.53	18.75
1.00	0.40	0.39	1.79	0.04	55.98	22.33	21.69
1.00	0.44	0.47	1.91	0.04	52.38	23.12	24.50
1.00	0.53	0.65	2.18	0.05	45.82	24.21	29.97
1.00	0.56	0.89	2.45	0.06	40.87	22.91	36.22
1.00	0.76	1.33	3.09	0.08	32.31	24.68	43.01

1.00	0.80	1.66	3.46	0.10	28.91	23.20	47.89

Table S12: EXSY data at 298 K

Relative integrals (o1p = -23.6)			Sum of integrals	Mixing time, d8 / s	Resonances as a % composition after mixing time		
-23.0	-23.6	4.64 (H_2)			-22.9	-23.5	4.64 (H_2)
0.13	1.00	0.08	1.21	0.01	10.94	82.69	6.38
0.24	1.00	0.19	1.42	0.01	16.70	70.28	13.02
0.34	1.00	0.32	1.66	0.02	20.43	60.22	19.36
0.47	1.00	0.51	1.98	0.02	23.57	50.61	25.82
0.52	1.00	0.68	2.20	0.03	23.82	45.46	30.71
0.64	1.00	0.90	2.54	0.04	25.12	39.33	35.55
0.70	1.00	1.42	3.11	0.05	22.39	32.12	45.49
**Relative integrals (o1p = -23.0)**			**Sum of integrals**	**Mixing time, d8 / s**	**Resonances as a % composition after mixing time**		
**-23.0**	**-23.6**	**4.64 (H_2)**			**-22.9**	**-23.5**	**4.64 (H_2)**
1.00	0.13	0.07	1.21	0.01	82.90	11.17	5.93
1.00	0.31	0.22	1.53	0.01	65.52	20.27	14.21
1.00	0.45	0.41	1.86	0.02	53.73	24.37	21.91
1.00	0.57	0.53	2.10	0.02	47.54	27.06	25.40
1.00	0.68	0.67	2.36	0.03	42.41	29.00	28.60
1.00	0.97	1.46	3.43	0.04	29.15	28.38	42.47
1.00	1.04	2.23	4.27	0.05	23.40	24.38	52.22

Analysis of these data yield rate constants for hydride site interchange and H_2 loss as detailed in Table S13.

Table S13. Hydride Ligand exchange are H_2 loss rates for **2**

Temperature	Rate (s^{-1}), H_2 loss	Hydride Site Interchange
263	0.14	0.00
268	0.50	0.38
273	0.59	0.60
278	0.90	1.34
283	1.55	3.09

288	2.93	6.53
293	6.64	9.97
298	13.96	26.98

Figure S2: Typical kinetic trace associated with the EXSY data used to determine rate constants for H_2 loss and H_a-H_b site interchange in **2**.

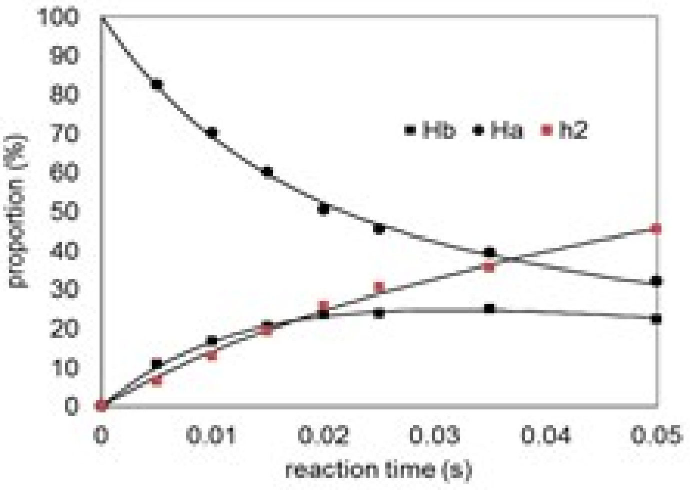

8. DFT calculations

All DFT calculations were performed on the full molecule (without simplification) using the Gaussian 09 software package.[2] Solvent effects were modelled using the integral equation formalism variant (IEFPCM) of the Polarizable Continuum Model (PCM)[3-5] with the solvent specified as methanol or dichloromethane as appropriate. Structures were optimised using the PBE0 functional of Adamo *et al.*[6] This hybrid functional has been shown to perform well for transition metals[7, 8] and a recent review of DFT by Tsipis recommends its use.[9] The def2-SVP basis set of Ahlrichs[10-13] was used for all atoms, except the hydride ligand atoms for which the def2-TZVPP basis set[10] was used, and iridium to which the LANL08(f) basis set was assigned together with the associated effective core potential (ECP).[14] Frequency calculations were used to identify local minima as well as first-order saddle points (transition states) and also provided zero-point energies and thermal corrections to the energy at 298.15 K. Thermodynamic parameters were determined from single point energy calculations performed on the optimised structures using the def2-TZVPP[10-13, 15] basis sets for all atoms, along with the GD3BJ empirical dispersion correction from Grimme which includes Beck-

Johnson damping[16]. The thermal energy corrections were then applied to obtain chemical enthalpies and free energies.

The effects of Basis Set Superposition Error (BSSE) were assessed by conducting counterpoise calculations[17, 18] on **2**. These calculations suggest that the magnitude of the errors arising for the Ir-N bond is small and so could reasonably be neglected (~ 4 kJ mol^{-1}) whereas the Ir-Cl bond has a larger error at 27 kJ mol^{-1} and so was included in the stability calculations.

The Free Energy profile for the catalytic cycle shown in Scheme 2 is shown below in Figure S3

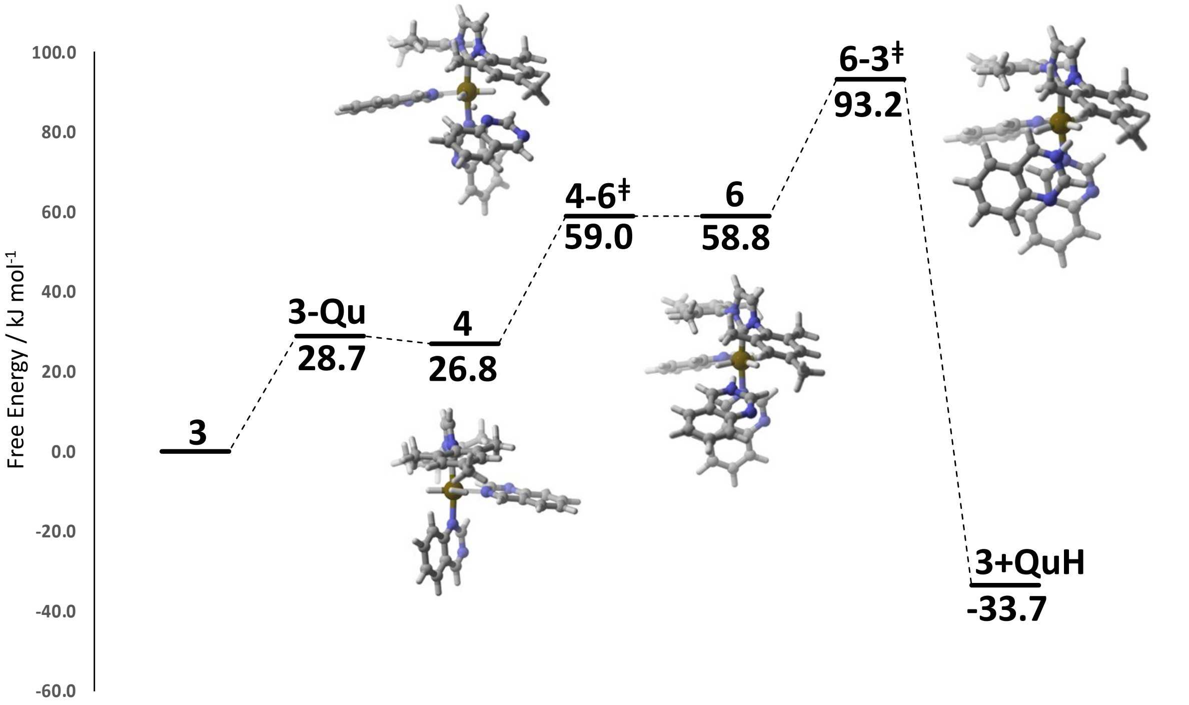

Figure S3: Catalytic cycle free energy profile for the hydrogenation of quinazoline into 3,4-dihydroquinazoline

It should be noted that free energy change for the loss of quinazoline is over-estimated by the calculations, arising from the creation of two species from one (**3**). The stabilisation of complex **4** from the coordination of dihydrogen is also underestimated for the same reason. The loss of protonated quinazoline from complex **6** is unfavourable, with the transition state **6**-3‡ lying on the lower energy pathway.

Table S14: Energy and Cartesian coordinates for **2** with DCM solvation

Sum of electronic and zero-point Energies= -2324.265187

Sum of electronic and thermal Free Energies= -2324.344957

Ir	0.683275000	0.222321000	-0.066846000
H	0.973143000	0.212945000	-1.615069000
H	2.186144000	0.614680000	0.145979000
C	1.596580000	-3.910755000	0.105682000
C	2.840568000	-3.384693000	0.077087000
H	1.252039000	-4.940201000	0.147231000
H	3.819039000	-3.856303000	0.092761000
C	1.368224000	-1.639831000	0.011330000
N	0.713092000	-2.844560000	0.067206000
N	2.692401000	-2.010309000	0.017975000
C	-0.697772000	-3.068892000	0.038109000
C	-1.383627000	-3.278148000	1.244133000
C	-1.334938000	-3.173130000	-1.207372000
C	-2.752878000	-3.554601000	1.174480000
C	-2.705362000	-3.445139000	-1.223244000
C	-3.434396000	-3.631102000	-0.043976000
H	-3.302442000	-3.716867000	2.106566000
H	-3.215974000	-3.517833000	-2.188002000
C	3.840352000	-1.157001000	-0.034878000
C	4.392086000	-0.838877000	-1.284139000
C	4.421566000	-0.726022000	1.166828000
C	5.543495000	-0.047013000	-1.309266000
C	5.572838000	0.062367000	1.088933000
C	6.149664000	0.413361000	-0.136072000
H	5.978163000	0.217899000	-2.277858000
H	6.030795000	0.415404000	2.017828000
C	-0.675025000	-3.204384000	2.563930000
H	-0.263583000	-2.193966000	2.728186000
H	-1.361291000	-3.442017000	3.388208000
H	0.169141000	-3.910582000	2.609470000
C	-0.560746000	-3.010941000	-2.482368000
H	-1.231332000	-3.003296000	-3.352156000
H	0.022888000	-2.077390000	-2.479230000
H	0.159326000	-3.834970000	-2.616494000
C	-4.914772000	-3.875758000	-0.084266000
H	-5.463612000	-2.919317000	-0.048074000
H	-5.215477000	-4.390952000	-1.007922000
H	-5.249095000	-4.476253000	0.774115000
C	3.802271000	-1.073956000	2.486572000
H	3.715895000	-2.164565000	2.617952000
H	4.401372000	-0.677175000	3.317515000
H	2.781632000	-0.659854000	2.557695000
C	3.747042000	-1.312533000	-2.552530000
H	2.723250000	-0.912828000	-2.635682000
H	4.320800000	-0.988843000	-3.431412000
H	3.665193000	-2.410697000	-2.581802000
C	7.405962000	1.235385000	-0.188225000
H	7.463684000	1.826507000	-1.113616000
H	7.472955000	1.923667000	0.666739000
H	8.299827000	0.589653000	-0.157311000

H	-6.852572000	-0.101970000	1.700652000
C	-6.080836000	-0.053555000	0.929240000
C	-6.459650000	0.091819000	-0.427699000
C	-4.750824000	-0.133923000	1.276294000
C	-5.512291000	0.156902000	-1.426389000
H	-7.520603000	0.153025000	-0.681973000
C	-3.760058000	-0.068798000	0.266528000
H	-4.446059000	-0.248061000	2.319557000
C	-4.138407000	0.079282000	-1.096067000
H	-5.788432000	0.270216000	-2.476424000
C	-2.372884000	-0.134475000	0.531175000
N	-3.191616000	0.151790000	-2.069533000
H	-1.983896000	-0.220549000	1.552586000
N	-1.473507000	-0.063814000	-0.431703000
C	-1.943964000	0.076003000	-1.708953000
H	-1.169696000	0.137216000	-2.478700000
H	-2.765940000	6.815487000	1.797485000
C	-2.084169000	6.276404000	1.136084000
C	-1.466289000	6.962685000	0.062897000
C	-1.829189000	4.940349000	1.348010000
C	-0.599484000	6.314678000	-0.790581000
H	-1.682244000	8.023290000	-0.086874000
C	-0.943157000	4.253727000	0.482200000
H	-2.299022000	4.399516000	2.173257000
C	-0.320847000	4.942215000	-0.595539000
H	-0.114650000	6.830589000	-1.621411000
C	-0.626718000	2.884478000	0.621029000
N	0.534063000	4.287377000	-1.426892000
H	-1.027652000	2.286277000	1.445116000
N	0.198096000	2.268715000	-0.209088000
C	0.752182000	3.025494000	-1.210884000
H	1.435578000	2.482388000	-1.868396000
Cl	0.273857000	0.345464000	2.424844000

Table S15: Energy and Cartesian coordinates for **3** with DCM solvation

Sum of electronic and zero-point Energies= -2281.555279

Sum of electronic and thermal Free Energies= -2281.6424

Ir	0.300265000	-0.033636000	-0.594537000
H	0.054050000	0.039430000	-2.154966000
H	1.825884000	-0.222110000	-0.888121000
C	0.901681000	-4.155314000	-1.254131000
C	-0.441014000	-4.189736000	-1.112102000
H	1.619257000	-4.941165000	-1.472258000
H	-1.149964000	-5.009998000	-1.178109000
C	0.214769000	-2.024011000	-0.810157000
N	1.289773000	-2.840907000	-1.070230000
N	-0.846160000	-2.894201000	-0.838983000
C	2.667577000	-2.467561000	-1.193291000
C	3.147441000	-2.051659000	-2.446676000
C	3.513179000	-2.606576000	-0.084617000
C	4.499707000	-1.722500000	-2.552002000

C	4.860657000	-2.262133000	-0.241246000
C	5.372872000	-1.813682000	-1.460988000
H	4.883657000	-1.386048000	-3.519731000
H	5.528674000	-2.352116000	0.620319000
C	-2.232264000	-2.592695000	-0.651953000
C	-2.845247000	-2.900239000	0.571726000
C	-2.969158000	-2.104652000	-1.746161000
C	-4.210278000	-2.619251000	0.709220000
C	-4.326949000	-1.844292000	-1.557722000
C	-4.964432000	-2.077500000	-0.332534000
H	-4.696780000	-2.842983000	1.663280000
H	-4.908429000	-1.455613000	-2.398655000
C	2.230997000	-1.952410000	-3.628618000
H	1.423190000	-1.229351000	-3.432368000
H	2.778800000	-1.632713000	-4.525134000
H	1.750076000	-2.919055000	-3.847934000
C	3.003993000	-3.130200000	1.225932000
H	2.842811000	-4.219966000	1.180555000
H	3.721289000	-2.933851000	2.034036000
H	2.039201000	-2.680517000	1.498420000
C	6.826095000	-1.467210000	-1.614770000
H	6.956841000	-0.526712000	-2.170937000
H	7.322814000	-1.364346000	-0.639986000
H	7.358788000	-2.249934000	-2.179692000
C	-2.324172000	-1.904913000	-3.085403000
H	-1.919160000	-2.854076000	-3.472404000
H	-3.048615000	-1.521212000	-3.815950000
H	-1.479129000	-1.202021000	-3.024040000
C	-2.115270000	-3.569650000	1.702381000
H	-1.026119000	-3.568986000	1.569773000
H	-2.353128000	-3.094517000	2.665683000
H	-2.428488000	-4.623329000	1.782976000
C	-6.414423000	-1.740608000	-0.143695000
H	-6.884528000	-2.384928000	0.612866000
H	-6.525955000	-0.698031000	0.199174000
H	-6.977028000	-1.836558000	-1.083399000
H	-6.400860000	1.674723000	3.054118000
C	-5.801277000	1.688658000	2.141502000
C	-6.293522000	2.359289000	0.994815000
C	-4.577854000	1.058985000	2.109978000
C	-5.565726000	2.397188000	-0.174656000
H	-7.267163000	2.852584000	1.043148000
C	-3.812000000	1.087307000	0.919073000
H	-4.188494000	0.538567000	2.988335000
C	-4.304718000	1.758951000	-0.233439000
H	-5.932291000	2.909767000	-1.065802000
C	-2.541797000	0.482671000	0.794570000
N	-3.568427000	1.789375000	-1.375360000
H	-2.110398000	-0.035358000	1.653812000
N	-1.838001000	0.530179000	-0.320269000
C	-2.413341000	1.193517000	-1.374059000
H	-1.811942000	1.214079000	-2.286365000
H	4.677283000	1.732198000	5.475443000

C	3.818683000	1.178766000	5.089407000
C	3.078404000	0.344547000	5.963195000
C	3.462993000	1.294042000	3.765559000
C	1.990318000	-0.370974000	5.513956000
H	3.380537000	0.269575000	7.010436000
C	2.345675000	0.569744000	3.280756000
H	4.026392000	1.934667000	3.083101000
C	1.603020000	-0.272391000	4.157512000
H	1.412972000	-1.019521000	6.175169000
C	1.905822000	0.624536000	1.942287000
N	0.540716000	-0.978729000	3.690975000
H	2.434852000	1.241375000	1.210219000
N	0.859277000	-0.063572000	1.514110000
C	0.228591000	-0.851352000	2.435453000
H	-0.607500000	-1.438520000	2.049930000
H	2.871019000	6.567831000	-3.431758000
C	2.330469000	6.052178000	-2.635284000
C	1.790406000	6.802809000	-1.561480000
C	2.177420000	4.686086000	-2.678562000
C	1.101629000	6.189612000	-0.538276000
H	1.924988000	7.886958000	-1.549331000
C	1.472487000	4.032330000	-1.636748000
H	2.589039000	4.097407000	-3.501728000
C	0.928787000	4.786106000	-0.556923000
H	0.680487000	6.755819000	0.294288000
C	1.265661000	2.639881000	-1.598512000
N	0.253123000	4.162575000	0.442339000
H	1.649556000	1.990976000	-2.388634000
N	0.599719000	2.055201000	-0.612423000
C	0.119737000	2.871263000	0.377900000
H	-0.420817000	2.366349000	1.183162000

Table S16: Energy and Cartesian coordinates for **2** with Methanol solvation

**Sum of electronic and zero-point Energies= -2324.269339**<br>**Sum of electronic and thermal Free Energies= -2324.349918**			
Ir	0.685207000	0.230847000	-0.068881000
H	0.959293000	0.217369000	-1.619170000
H	2.188819000	0.634697000	0.126877000
C	1.622551000	-3.898471000	0.097989000
C	2.864013000	-3.366340000	0.077907000
H	1.283162000	-4.929844000	0.134145000
H	3.844559000	-3.833552000	0.096771000
C	1.383046000	-1.628565000	0.009135000
N	0.733941000	-2.836549000	0.057754000
N	2.709169000	-1.992371000	0.022333000
C	-0.675460000	-3.070137000	0.018844000
C	-1.366794000	-3.290689000	1.219748000
C	-1.304413000	-3.172765000	-1.230806000
C	-2.732851000	-3.579534000	1.141185000
C	-2.672501000	-3.456488000	-1.255794000
C	-3.406570000	-3.655779000	-0.081836000

H	-3.286550000	-3.750687000	2.069194000
H	-3.177025000	-3.528574000	-2.223795000
C	3.853363000	-1.133975000	-0.026054000
C	4.405996000	-0.810594000	-1.273889000
C	4.431108000	-0.703002000	1.177307000
C	5.550633000	-0.008660000	-1.295694000
C	5.575849000	0.095726000	1.103030000
C	6.150887000	0.455260000	-0.120482000
H	5.985546000	0.260383000	-2.263025000
H	6.031099000	0.448478000	2.033385000
C	-0.666894000	-3.214338000	2.544178000
H	-0.261168000	-2.202254000	2.711256000
H	-1.357367000	-3.455064000	3.363970000
H	0.179925000	-3.917034000	2.595034000
C	-0.524776000	-2.996009000	-2.500469000
H	-1.189737000	-2.997552000	-3.374565000
H	0.043401000	-2.052923000	-2.491664000
H	0.209407000	-3.807954000	-2.631398000
C	-4.884415000	-3.913487000	-0.131348000
H	-5.442270000	-2.963428000	-0.072104000
H	-5.178522000	-4.408311000	-1.068094000
H	-5.214588000	-4.537678000	0.711614000
C	3.816985000	-1.063922000	2.496214000
H	3.751161000	-2.156268000	2.625223000
H	4.409028000	-0.658651000	3.328164000
H	2.789048000	-0.668827000	2.567943000
C	3.770547000	-1.291652000	-2.544457000
H	2.738490000	-0.914142000	-2.627907000
H	4.338285000	-0.953334000	-3.421673000
H	3.712057000	-2.391212000	-2.577576000
C	7.400323000	1.287774000	-0.169786000
H	7.442406000	1.899285000	-1.082739000
H	7.473817000	1.956790000	0.699706000
H	8.298730000	0.647735000	-0.165335000
H	-6.835674000	-0.188638000	1.746503000
C	-6.069053000	-0.121761000	0.971445000
C	-6.457379000	0.041443000	-0.380772000
C	-4.736333000	-0.196428000	1.309376000
C	-5.516804000	0.129734000	-1.384213000
H	-7.520324000	0.097603000	-0.627611000
C	-3.752565000	-0.107609000	0.294644000
H	-4.424536000	-0.323932000	2.348944000
C	-4.140510000	0.058195000	-1.063257000
H	-5.801294000	0.256746000	-2.430457000
C	-2.363432000	-0.165222000	0.549949000
N	-3.199811000	0.152820000	-2.041019000
H	-1.969827000	-0.262217000	1.568203000
N	-1.470119000	-0.072092000	-0.416779000
C	-1.949421000	0.081902000	-1.689249000
H	-1.180610000	0.160020000	-2.462910000
H	-2.867198000	6.778765000	1.763699000
C	-2.170787000	6.249233000	1.109951000
C	-1.547934000	6.945184000	0.045830000

C	-1.902812000	4.915723000	1.322328000
C	-0.663444000	6.309359000	-0.798837000
H	-1.774671000	8.003434000	-0.104328000
C	-0.998036000	4.241790000	0.465897000
H	-2.376904000	4.367489000	2.140161000
C	-0.371302000	4.939910000	-0.603299000
H	-0.175699000	6.833578000	-1.622765000
C	-0.665851000	2.876624000	0.606688000
N	0.500397000	4.296145000	-1.426241000
H	-1.070866000	2.273627000	1.424913000
N	0.176620000	2.272079000	-0.214268000
C	0.730933000	3.036544000	-1.209834000
H	1.426562000	2.502647000	-1.862087000
Cl	0.293666000	0.360832000	2.429143000

Table S17: Energy and Cartesian coordinates for **3** with Methanol solvation

**Sum of electronic and zero-point Energies= -2281.56098**			
**Sum of electronic and thermal Free Energies= -2281.648747**			
Ir	0.300265000	-0.033636000	-0.594537000
H	0.054050000	0.039430000	-2.154966000
H	1.825884000	-0.222110000	-0.888121000
C	0.901681000	-4.155314000	-1.254131000
C	-0.441014000	-4.189736000	-1.112102000
H	1.619257000	-4.941165000	-1.472258000
H	-1.149964000	-5.009998000	-1.178109000
C	0.214769000	-2.024011000	-0.810157000
N	1.289773000	-2.840907000	-1.070230000
N	-0.846160000	-2.894201000	-0.838983000
C	2.667577000	-2.467561000	-1.193291000
C	3.147441000	-2.051659000	-2.446676000
C	3.513179000	-2.606576000	-0.084617000
C	4.499707000	-1.722500000	-2.552002000
C	4.860657000	-2.262133000	-0.241246000
C	5.372872000	-1.813682000	-1.460988000
H	4.883657000	-1.386048000	-3.519731000
H	5.528674000	-2.352116000	0.620319000
C	-2.232264000	-2.592695000	-0.651953000
C	-2.845247000	-2.900239000	0.571726000
C	-2.969158000	-2.104652000	-1.746161000
C	-4.210278000	-2.619251000	0.709220000
C	-4.326949000	-1.844292000	-1.557722000
C	-4.964432000	-2.077500000	-0.332534000
H	-4.696780000	-2.842983000	1.663280000
H	-4.908429000	-1.455613000	-2.398655000
C	2.230997000	-1.952410000	-3.628618000
H	1.423190000	-1.229351000	-3.432368000
H	2.778800000	-1.632713000	-4.525134000
H	1.750076000	-2.919055000	-3.847934000
C	3.003993000	-3.130200000	1.225932000
H	2.842811000	-4.219966000	1.180555000
H	3.721289000	-2.933851000	2.034036000

H	2.039201000	-2.680517000	1.498420000
C	6.826095000	-1.467210000	-1.614770000
H	6.956841000	-0.526712000	-2.170937000
H	7.322814000	-1.364346000	-0.639986000
H	7.358788000	-2.249934000	-2.179692000
C	-2.324172000	-1.904913000	-3.085403000
H	-1.919160000	-2.854076000	-3.472404000
H	-3.048615000	-1.521212000	-3.815950000
H	-1.479129000	-1.202021000	-3.024040000
C	-2.115270000	-3.569650000	1.702381000
H	-1.026119000	-3.568986000	1.569773000
H	-2.353128000	-3.094517000	2.665683000
H	-2.428488000	-4.623329000	1.782976000
C	-6.414423000	-1.740608000	-0.143695000
H	-6.884528000	-2.384928000	0.612866000
H	-6.525955000	-0.698031000	0.199174000
H	-6.977028000	-1.836558000	-1.083399000
H	-6.400860000	1.674723000	3.054118000
C	-5.801277000	1.688658000	2.141502000
C	-6.293522000	2.359289000	0.994815000
C	-4.577854000	1.058985000	2.109978000
C	-5.565726000	2.397188000	-0.174656000
H	-7.267163000	2.852584000	1.043148000
C	-3.812000000	1.087307000	0.919073000
H	-4.188494000	0.538567000	2.988335000
C	-4.304718000	1.758951000	-0.233439000
H	-5.932291000	2.909767000	-1.065802000
C	-2.541797000	0.482671000	0.794570000
N	-3.568427000	1.789375000	-1.375360000
H	-2.110398000	-0.035358000	1.653812000
N	-1.838001000	0.530179000	-0.320269000
C	-2.413341000	1.193517000	-1.374059000
H	-1.811942000	1.214079000	-2.286365000
H	4.677283000	1.732198000	5.475443000
C	3.818683000	1.178766000	5.089407000
C	3.078404000	0.344547000	5.963195000
C	3.462993000	1.294042000	3.765559000
C	1.990318000	-0.370974000	5.513956000
H	3.380537000	0.269575000	7.010436000
C	2.345675000	0.569744000	3.280756000
H	4.026392000	1.934667000	3.083101000
C	1.603020000	-0.272391000	4.157512000
H	1.412972000	-1.019521000	6.175169000
C	1.905822000	0.624536000	1.942287000
N	0.540716000	-0.978729000	3.690975000
H	2.434852000	1.241375000	1.210219000
N	0.859277000	-0.063572000	1.514110000
C	0.228591000	-0.851352000	2.435453000
H	-0.607500000	-1.438520000	2.049930000
H	2.871019000	6.567831000	-3.431758000
C	2.330469000	6.052178000	-2.635284000
C	1.790406000	6.802809000	-1.561480000
C	2.177420000	4.686086000	-2.678562000

C	1.101629000	6.189612000	-0.538276000
H	1.924988000	7.886958000	-1.549331000
C	1.472487000	4.032330000	-1.636748000
H	2.589039000	4.097407000	-3.501728000
C	0.928787000	4.786106000	-0.556923000
H	0.680487000	6.755819000	0.294288000
C	1.265661000	2.639881000	-1.598512000
N	0.253123000	4.162575000	0.442339000
H	1.649556000	1.990976000	-2.388634000
N	0.599719000	2.055201000	-0.612423000
C	0.119737000	2.871263000	0.377900000
H	-0.420817000	2.366349000	1.183162000

Catalytic cycle:

Table S18: Energy and Cartesian coordinates for **3-Qu**

**Sum of electronic and zero-point Energies= -1864.001798**<br>**Sum of electronic and thermal Free Energies= -1864.080331**			
Ir	-0.031570000	-0.394814000	0.644663000
H	1.258001000	-0.410462000	1.464090000
H	-0.431439000	-1.716594000	1.424298000
C	2.262256000	-2.901467000	-1.853553000
C	1.023216000	-3.097962000	-2.363163000
H	3.222842000	-3.330272000	-2.124545000
H	0.674385000	-3.732438000	-3.173216000
C	0.834970000	-1.594753000	-0.669785000
N	2.134180000	-1.981707000	-0.823893000
N	0.165164000	-2.295248000	-1.632612000
C	3.246334000	-1.498042000	-0.064673000
C	4.058666000	-0.496316000	-0.617561000
C	3.519522000	-2.070689000	1.189088000
C	5.138795000	-0.036123000	0.143414000
C	4.609926000	-1.575676000	1.908061000
C	5.425283000	-0.552698000	1.409872000
H	5.775178000	0.750872000	-0.271529000
H	4.832358000	-2.009228000	2.887460000
C	-1.248254000	-2.200147000	-1.841686000
C	-2.095331000	-3.087632000	-1.156157000
C	-1.743576000	-1.237590000	-2.736374000
C	-3.472349000	-2.960663000	-1.358105000
C	-3.129525000	-1.150535000	-2.899971000
C	-4.010121000	-1.994030000	-2.215030000
H	-4.144755000	-3.644096000	-0.831550000
H	-3.530372000	-0.404318000	-3.592046000
C	3.806885000	0.052024000	-1.992613000
H	2.733917000	0.160970000	-2.205813000
H	4.285305000	1.032777000	-2.116696000
H	4.218217000	-0.618905000	-2.764852000
C	2.675213000	-3.185413000	1.731517000
H	3.069834000	-3.543722000	2.691562000
H	1.635489000	-2.854572000	1.881964000

H	2.642934000	-4.037409000	1.034008000
C	6.565695000	-0.013802000	2.224409000
H	6.211617000	0.760226000	2.925700000
H	7.038991000	-0.802944000	2.826521000
H	7.334328000	0.447634000	1.588480000
C	-0.818549000	-0.365826000	-3.534703000
H	-0.354271000	-0.938692000	-4.354348000
H	-1.361089000	0.476526000	-3.984312000
H	0.003485000	0.034287000	-2.924299000
C	-1.539117000	-4.152399000	-0.257721000
H	-0.893489000	-3.717205000	0.520135000
H	-2.346864000	-4.711376000	0.232906000
H	-0.924759000	-4.870286000	-0.824443000
C	-5.495496000	-1.854296000	-2.381934000
H	-6.015189000	-2.801025000	-2.177341000
H	-5.896287000	-1.102385000	-1.681535000
H	-5.758390000	-1.524017000	-3.397147000
H	-1.498335000	5.910540000	-3.490791000
C	-0.775978000	5.335282000	-2.907876000
C	0.534272000	5.842679000	-2.726002000
C	-1.139207000	4.128290000	-2.356881000
C	1.473869000	5.147254000	-1.996834000
H	0.799499000	6.803265000	-3.173844000
C	-0.188519000	3.394374000	-1.605210000
H	-2.146522000	3.726360000	-2.489771000
C	1.129257000	3.903007000	-1.419655000
H	2.486066000	5.527560000	-1.848745000
C	-0.462193000	2.146557000	-1.009713000
N	2.042898000	3.202733000	-0.697276000
H	-1.452950000	1.690830000	-1.106727000
N	0.449054000	1.480357000	-0.321290000
C	1.681970000	2.059695000	-0.193826000
H	2.410552000	1.484146000	0.384647000
H	-6.246986000	0.393186000	2.980286000
C	-5.171039000	0.469961000	2.811428000
C	-4.557559000	-0.337951000	1.826065000
C	-4.411306000	1.342183000	3.554726000
C	-3.202439000	-0.270368000	1.583313000
H	-5.168514000	-1.034807000	1.247337000
C	-3.016369000	1.432103000	3.326950000
H	-4.864063000	1.971495000	4.324499000
C	-2.401295000	0.624180000	2.329328000
H	-2.744700000	-0.917014000	0.830940000
C	-2.169827000	2.301559000	4.051949000
N	-1.046610000	0.731178000	2.116108000
H	-2.591793000	2.947734000	4.831149000
N	-0.877191000	2.372992000	3.831327000
C	-0.372665000	1.585774000	2.872190000
H	0.703726000	1.648630000	2.692246000

Table S18: Energy and Cartesian coordinates for **4**

**Sum of electronic and zero-point Energies= -2282.71586**

Sum of electronic and thermal Free Energies= -2282.808754			
Ir	0.206752000	-0.187560000	-0.633328000
H	-1.180942000	-0.162489000	-1.404670000
H	0.633243000	-1.234134000	-1.737969000
C	-1.783471000	-3.461448000	1.160610000
C	-0.563095000	-3.608307000	1.723045000
H	-2.691266000	-4.050522000	1.253364000
H	-0.174508000	-4.353076000	2.411769000
C	-0.483304000	-1.760620000	0.398734000
N	-1.723554000	-2.335370000	0.357882000
N	0.217878000	-2.568766000	1.251899000
C	-2.872012000	-1.892284000	-0.375007000
C	-3.874239000	-1.185349000	0.304603000
C	-3.005378000	-2.260323000	-1.725731000
C	-4.998400000	-0.779925000	-0.425759000
C	-4.146558000	-1.835000000	-2.406141000
C	-5.148685000	-1.082176000	-1.780245000
H	-5.781969000	-0.215788000	0.088312000
H	-4.260949000	-2.108537000	-3.459166000
C	1.589999000	-2.418855000	1.629917000
C	2.578276000	-3.039670000	0.851218000
C	1.899649000	-1.675071000	2.779058000
C	3.912076000	-2.864509000	1.232803000
C	3.247528000	-1.530389000	3.116898000
C	4.268261000	-2.112751000	2.356933000
H	4.695555000	-3.334416000	0.631360000
H	3.506743000	-0.945281000	4.004042000
C	-3.787927000	-0.901448000	1.777187000
H	-2.751374000	-0.789265000	2.123417000
H	-4.339091000	0.013623000	2.033266000
H	-4.231866000	-1.727895000	2.356834000
C	-1.964166000	-3.094771000	-2.408498000
H	-2.278281000	-3.355425000	-3.427832000
H	-1.005140000	-2.556785000	-2.465569000
H	-1.775049000	-4.029677000	-1.857515000
C	-6.348239000	-0.616576000	-2.553707000
H	-6.077622000	0.203040000	-3.239435000
H	-6.764413000	-1.426435000	-3.171724000
H	-7.140718000	-0.246858000	-1.888477000
C	0.819506000	-1.062524000	3.621428000
H	0.191926000	-1.837391000	4.090273000
H	1.248258000	-0.446265000	4.422496000
H	0.145382000	-0.431060000	3.023190000
C	2.217321000	-3.859979000	-0.352299000
H	1.681815000	-3.255204000	-1.101250000
H	3.116077000	-4.277400000	-0.824883000
H	1.553209000	-4.696443000	-0.083141000
C	5.706072000	-1.963348000	2.762262000
H	6.383990000	-2.113304000	1.910296000
H	5.901733000	-0.970868000	3.193459000
H	5.972972000	-2.708387000	3.530261000
H	0.409322000	5.460962000	4.661325000
C	-0.182713000	4.845927000	3.980464000

C	-1.581549000	5.053540000	3.893101000
C	0.428530000	3.879042000	3.216522000
C	-2.363746000	4.299399000	3.046087000
H	-2.043814000	5.827324000	4.510482000
C	-0.356228000	3.090704000	2.338735000
H	1.506200000	3.709172000	3.275463000
C	-1.762113000	3.298889000	2.248996000
H	-3.441868000	4.449824000	2.968640000
C	0.178509000	2.074623000	1.521142000
N	-2.513626000	2.545979000	1.404614000
H	1.251789000	1.867000000	1.542165000
N	-0.576720000	1.347708000	0.712683000
C	-1.916897000	1.635392000	0.695734000
H	-2.513257000	1.025997000	0.011779000
H	5.581175000	1.836870000	-4.281079000
C	4.603966000	1.752274000	-3.801692000
C	4.283245000	0.594234000	-3.058522000
C	3.676069000	2.758219000	-3.927248000
C	3.062040000	0.451992000	-2.434646000
H	5.016327000	-0.212030000	-2.980996000
C	2.411276000	2.634407000	-3.302763000
H	3.891046000	3.657369000	-4.509328000
C	2.095694000	1.478826000	-2.528608000
H	2.824254000	-0.460547000	-1.892680000
C	1.407349000	3.619712000	-3.422889000
N	0.863076000	1.394700000	-1.919130000
H	1.603196000	4.530459000	-4.001560000
N	0.223842000	3.486214000	-2.871602000
C	0.006732000	2.380492000	-2.149430000
H	-0.983808000	2.277842000	-1.702784000
H	1.636179000	0.019923000	0.359789000
H	1.809085000	-0.639136000	-0.173793000

Table S19: Energy and Cartesian coordinates for **4-6‡**

**Sum of electronic and zero-point Energies= -2282.706605**			
**Sum of electronic and thermal Free Energies= -2282.797819**			
Ir	0.201273000	-0.177697000	-0.658899000
H	-0.943934000	-0.590605000	-1.667329000
H	1.165939000	-0.053256000	-1.941827000
C	1.686342000	-4.135373000	-0.671481000
C	0.366646000	-4.388078000	-0.529390000
H	2.539980000	-4.802359000	-0.752300000
H	-0.181566000	-5.323511000	-0.462397000
C	0.618818000	-2.123389000	-0.584866000
N	1.827611000	-2.757782000	-0.704858000
N	-0.271069000	-3.159632000	-0.476057000
C	3.115379000	-2.142844000	-0.800179000
C	3.619657000	-1.793198000	-2.062330000
C	3.879175000	-1.999375000	0.369643000
C	4.890420000	-1.212706000	-2.118793000
C	5.146420000	-1.421433000	0.258557000

C	5.662622000	-1.003583000	-0.971795000
H	5.291891000	-0.924674000	-3.094848000
H	5.746299000	-1.291792000	1.164194000
C	-1.690610000	-3.067851000	-0.323024000
C	-2.232166000	-3.048583000	0.970136000
C	-2.503060000	-3.102011000	-1.468113000
C	-3.625505000	-3.026205000	1.098088000
C	-3.887892000	-3.076236000	-1.287517000
C	-4.470208000	-3.037650000	-0.014711000
H	-4.060105000	-3.003841000	2.101677000
H	-4.532600000	-3.094125000	-2.171231000
C	2.840236000	-2.069223000	-3.313635000
H	1.860998000	-1.567910000	-3.286850000
H	3.387332000	-1.722423000	-4.200533000
H	2.649600000	-3.148196000	-3.431390000
C	3.376430000	-2.490246000	1.696398000
H	3.508542000	-3.581506000	1.785075000
H	3.927108000	-2.022789000	2.523904000
H	2.304186000	-2.290726000	1.832728000
C	7.000124000	-0.326838000	-1.051038000
H	7.431333000	-0.395410000	-2.059730000
H	6.902098000	0.743840000	-0.804800000
H	7.715160000	-0.758855000	-0.335615000
C	-1.903131000	-3.180155000	-2.840724000
H	-1.316929000	-4.104139000	-2.969675000
H	-2.684382000	-3.164165000	-3.612326000
H	-1.216975000	-2.337806000	-3.019434000
C	-1.346140000	-3.071677000	2.180624000
H	-0.605982000	-2.257287000	2.147612000
H	-1.935424000	-2.968920000	3.101518000
H	-0.779594000	-4.014859000	2.243721000
C	-5.963979000	-3.034557000	0.140405000
H	-6.434747000	-2.318973000	-0.550617000
H	-6.385358000	-4.027084000	-0.089287000
H	-6.264073000	-2.774251000	1.165007000
H	-1.087299000	6.223161000	-4.644117000
C	-1.059717000	5.726220000	-3.672092000
C	-1.391645000	6.458966000	-2.505790000
C	-0.704771000	4.399976000	-3.585482000
C	-1.367968000	5.867601000	-1.261700000
H	-1.670178000	7.511261000	-2.599552000
C	-0.674268000	3.769900000	-2.316321000
H	-0.445906000	3.824516000	-4.477478000
C	-1.007014000	4.505426000	-1.143379000
H	-1.620897000	6.420650000	-0.355396000
C	-0.321254000	2.417922000	-2.132178000
N	-0.977331000	3.903393000	0.074202000
H	-0.047205000	1.786921000	-2.980354000
N	-0.304240000	1.853076000	-0.934520000
C	-0.642121000	2.649300000	0.128319000
H	-0.618322000	2.156810000	1.104548000
H	3.825957000	1.783348000	5.887550000
C	3.808620000	1.786279000	4.795638000

C	4.833176000	2.457719000	4.084388000
C	2.797472000	1.142800000	4.119595000
C	4.845283000	2.482833000	2.706796000
H	5.625453000	2.962897000	4.641928000
C	2.790010000	1.155303000	2.703010000
H	2.000530000	0.622889000	4.656814000
C	3.820047000	1.826822000	1.986238000
H	5.629031000	2.996207000	2.146787000
C	1.790131000	0.534032000	1.924434000
N	3.818399000	1.836052000	0.627160000
H	0.954866000	0.016381000	2.404278000
N	1.810717000	0.554828000	0.603683000
C	2.857706000	1.213098000	0.011623000
H	2.850728000	1.193939000	-1.080895000
H	-0.734300000	-0.336769000	0.769785000
H	-1.663611000	-0.064540000	0.371893000
H	-5.729074000	1.385232000	4.693071000
C	-5.165609000	1.155943000	3.786439000
C	-3.788678000	0.843532000	3.882988000
C	-5.790308000	1.171568000	2.560484000
C	-3.038391000	0.547623000	2.764895000
H	-3.311816000	0.836819000	4.865770000
C	-5.046117000	0.872275000	1.394442000
H	-6.852222000	1.411583000	2.469621000
C	-3.663058000	0.558259000	1.498272000
H	-1.978242000	0.304781000	2.845953000
C	-5.594839000	0.863252000	0.088692000
N	-2.973207000	0.276482000	0.358621000
H	-6.656644000	1.101424000	-0.049731000
N	-4.891292000	0.580002000	-0.985121000
C	-3.591976000	0.291710000	-0.798497000
H	-2.980561000	0.052970000	-1.674703000

Table S20: Energy and Cartesian coordinates for **6**

Sum of electronic and zero-point Energies= -2282.707802

Sum of electronic and thermal Free Energies= -2282.794284

Ir	0.195200000	-0.412116000	-0.629973000
H	0.569874000	-0.807612000	-2.186874000
C	-0.129125000	-4.544816000	0.140934000
C	1.156577000	-4.377020000	0.516168000
H	-0.757397000	-5.430507000	0.110523000
H	1.896561000	-5.082017000	0.884252000
C	0.371278000	-2.329536000	-0.131998000
N	-0.598692000	-3.300593000	-0.247276000
N	1.446282000	-3.031451000	0.352254000
C	-1.965488000	-3.129522000	-0.630362000
C	-2.928167000	-2.964909000	0.379248000
C	-2.319819000	-3.214361000	-1.983921000
C	-4.265827000	-2.838551000	-0.003161000
C	-3.671479000	-3.074131000	-2.317162000
C	-4.657914000	-2.877373000	-1.346416000
H	-5.024102000	-2.701417000	0.773667000

H	-3.960043000	-3.124839000	-3.371160000
C	2.724676000	-2.511557000	0.718731000
C	3.763584000	-2.529644000	-0.221912000
C	2.922491000	-2.077939000	2.038251000
C	5.016906000	-2.057362000	0.179265000
C	4.195387000	-1.624445000	2.394126000
C	5.252479000	-1.595618000	1.478084000
H	5.834369000	-2.053500000	-0.547695000
H	4.363629000	-1.278527000	3.418313000
C	-2.531814000	-2.929763000	1.826525000
H	-1.697619000	-2.232312000	1.998457000
H	-3.377969000	-2.627451000	2.458510000
H	-2.191175000	-3.919454000	2.172112000
C	-1.276508000	-3.435856000	-3.036370000
H	-0.738376000	-4.383403000	-2.873087000
H	-1.726148000	-3.466229000	-4.038007000
H	-0.526474000	-2.628919000	-3.005574000
C	-6.099699000	-2.693317000	-1.723616000
H	-6.427245000	-1.662397000	-1.510580000
H	-6.266859000	-2.886441000	-2.792252000
H	-6.754367000	-3.363445000	-1.145462000
C	1.798127000	-2.095291000	3.031765000
H	1.405459000	-3.114211000	3.178634000
H	2.129689000	-1.714115000	4.006894000
H	0.955542000	-1.476877000	2.682032000
C	3.538801000	-3.054805000	-1.609710000
H	2.633994000	-2.622231000	-2.063521000
H	4.397499000	-2.833958000	-2.257895000
H	3.397782000	-4.148242000	-1.600299000
C	6.595167000	-1.050150000	1.869056000
H	7.406895000	-1.526802000	1.300907000
H	6.645495000	0.033056000	1.665129000
H	6.791645000	-1.189191000	2.941872000
H	-0.602365000	7.190594000	0.042488000
C	-0.616635000	6.307272000	-0.599514000
C	-0.894289000	6.454449000	-1.980410000
C	-0.365991000	5.063208000	-0.066925000
C	-0.920743000	5.362945000	-2.821376000
H	-1.090210000	7.451731000	-2.381339000
C	-0.386738000	3.927313000	-0.913134000
H	-0.149795000	4.937711000	0.996832000
C	-0.666372000	4.073451000	-2.300397000
H	-1.133103000	5.461634000	-3.887442000
C	-0.143989000	2.613782000	-0.457694000
N	-0.686947000	2.984051000	-3.113897000
H	0.067515000	2.418566000	0.596823000
N	-0.168478000	1.568427000	-1.268717000
C	-0.444565000	1.820228000	-2.591023000
H	-0.448140000	0.933829000	-3.229361000
H	5.821482000	3.507712000	2.890431000
C	5.458048000	3.013951000	1.986614000
C	6.201917000	3.121783000	0.786372000
C	4.285348000	2.293223000	2.017111000

C	5.773814000	2.510436000	-0.372347000
H	7.129694000	3.698864000	0.783765000
C	3.825930000	1.658925000	0.837575000
H	3.702872000	2.202889000	2.937259000
C	4.572403000	1.763431000	-0.367747000
H	6.337328000	2.584264000	-1.304321000
C	2.629337000	0.909502000	0.771752000
N	4.136113000	1.152308000	-1.501448000
H	1.993015000	0.792682000	1.652420000
N	2.217289000	0.334956000	-0.343954000
C	3.023273000	0.482437000	-1.444076000
H	2.655121000	-0.014802000	-2.344732000
H	-0.163435000	0.045313000	0.953084000
H	-1.283768000	-0.815328000	-1.006026000
H	-5.972761000	2.698756000	4.975959000
C	-5.743929000	2.311687000	3.981211000
C	-6.770303000	2.244839000	3.006905000
C	-4.469480000	1.893841000	3.680588000
C	-6.527311000	1.764531000	1.738212000
H	-7.775028000	2.582902000	3.270919000
C	-4.194377000	1.394151000	2.383052000
H	-3.668247000	1.939696000	4.421306000
C	-5.229976000	1.326413000	1.395397000
H	-7.311596000	1.710432000	0.981794000
C	-2.928266000	0.942186000	1.985237000
N	-4.984327000	0.850137000	0.144496000
H	-2.053283000	0.939962000	2.641810000
N	-2.757429000	0.488873000	0.758121000
C	-3.784726000	0.450038000	-0.134865000
H	-3.535808000	0.052588000	-1.122951000
H	-1.789263000	0.138662000	0.454970000

Table S21: Energy and Cartesian coordinates for **6-3‡**

**Sum of electronic and zero-point Energies= -2282.694295**<br>**Sum of electronic and thermal Free Energies= -2282.783452**			
Ir	-0.392578000	-0.519737000	-0.596775000
H	-0.318108000	-0.993748000	-2.172081000
C	-1.451868000	-4.499706000	0.296248000
C	-0.112348000	-4.627249000	0.405116000
H	-2.251645000	-5.227742000	0.398421000
H	0.512069000	-5.489503000	0.621530000
C	-0.539487000	-2.433522000	-0.090548000
N	-1.703743000	-3.170263000	0.000144000
N	0.427936000	-3.371226000	0.176742000
C	-3.041013000	-2.677726000	-0.087317000
C	-3.718882000	-2.364956000	1.100594000
C	-3.662079000	-2.572403000	-1.342423000
C	-5.024363000	-1.863157000	1.002175000
C	-4.966545000	-2.076144000	-1.389341000
C	-5.662696000	-1.704033000	-0.232966000
H	-5.561185000	-1.606305000	1.920482000

H	-5.454570000	-1.974948000	-2.363217000
C	1.834262000	-3.151393000	0.288614000
C	2.642324000	-3.296543000	-0.846590000
C	2.372410000	-2.877397000	1.555493000
C	4.021654000	-3.122457000	-0.694324000
C	3.756777000	-2.722482000	1.658962000
C	4.597845000	-2.830745000	0.545682000
H	4.664140000	-3.221517000	-1.574146000
H	4.190537000	-2.504734000	2.639497000
C	-3.083946000	-2.589543000	2.443684000
H	-2.015453000	-2.328161000	2.445322000
H	-3.596397000	-2.014295000	3.227290000
H	-3.143225000	-3.652844000	2.728811000
C	-2.929456000	-2.961373000	-2.589007000
H	-2.603824000	-4.013010000	-2.548563000
H	-3.562169000	-2.831019000	-3.477136000
H	-2.021269000	-2.345732000	-2.699860000
C	-7.070755000	-1.189946000	-0.317437000
H	-7.375039000	-0.688098000	0.611581000
H	-7.190281000	-0.481836000	-1.150780000
H	-7.777593000	-2.017362000	-0.494611000
C	1.485231000	-2.745232000	2.758922000
H	0.913523000	-3.668411000	2.944363000
H	2.074348000	-2.523152000	3.658703000
H	0.747321000	-1.938352000	2.619605000
C	2.039513000	-3.628563000	-2.179355000
H	1.240650000	-2.915380000	-2.438243000
H	2.799782000	-3.610430000	-2.971690000
H	1.582036000	-4.631344000	-2.170838000
C	6.075903000	-2.604926000	0.678299000
H	6.633915000	-3.080615000	-0.140536000
H	6.305179000	-1.526058000	0.651468000
H	6.459459000	-2.993237000	1.633222000
H	1.002854000	7.032762000	-0.542493000
C	0.538112000	6.174439000	-1.032403000
C	-0.173300000	6.363872000	-2.241824000
C	0.646281000	4.920490000	-0.474777000
C	-0.769733000	5.303159000	-2.888979000
H	-0.246991000	7.368168000	-2.665666000
C	0.041025000	3.815735000	-1.122644000
H	1.192573000	4.764268000	0.458400000
C	-0.671471000	4.004447000	-2.339357000
H	-1.319631000	5.433101000	-3.822852000
C	0.095607000	2.494880000	-0.626414000
N	-1.248665000	2.944697000	-2.967140000
H	0.610154000	2.274386000	0.312669000
N	-0.465177000	1.478241000	-1.259907000
C	-1.124260000	1.769320000	-2.430307000
H	-1.569794000	0.904027000	-2.926331000
H	6.390035000	1.898255000	2.193507000
C	5.801618000	1.572802000	1.332894000
C	6.364760000	1.647366000	0.035440000
C	4.522900000	1.094801000	1.511403000

C	5.651693000	1.243706000	-1.072660000
H	7.381010000	2.030509000	-0.083868000
C	3.771494000	0.676192000	0.386421000
H	4.078605000	1.032749000	2.507903000
C	4.335960000	0.747308000	-0.916763000
H	6.073066000	1.294204000	-2.078390000
C	2.448841000	0.185564000	0.470933000
N	3.618933000	0.343243000	-1.999047000
H	1.937653000	0.121669000	1.435020000
N	1.765790000	-0.191597000	-0.594748000
C	2.412940000	-0.101323000	-1.800079000
H	1.821428000	-0.432239000	-2.657594000
H	-0.508963000	0.066983000	0.988919000
H	-1.962354000	-0.571175000	-0.678001000
H	0.807656000	3.401618000	4.688860000
C	0.009538000	3.370208000	3.944606000
C	-0.491949000	4.577046000	3.407626000
C	-0.504907000	2.159533000	3.534321000
C	-1.500863000	4.574628000	2.464801000
H	-0.071837000	5.527089000	3.745685000
C	-1.540207000	2.135951000	2.572985000
H	-0.125870000	1.218547000	3.939372000
C	-2.047564000	3.351368000	2.027637000
H	-1.895656000	5.499763000	2.042225000
C	-2.127380000	0.934623000	2.113020000
N	-3.057197000	3.355639000	1.101956000
H	-1.912581000	-0.035441000	2.550491000
N	-3.162021000	1.028321000	1.277907000
C	-3.573640000	2.224115000	0.762506000
H	-4.391423000	2.171052000	0.038216000
H	-3.612461000	0.173491000	0.958460000

Table S22: Energy and Cartesian coordinates for **5**

**Sum of electronic and zero-point Energies= -2285.110372**			
**Sum of electronic and thermal Free Energies= -2285.199016**			
Ir	-0.392578000	-0.519737000	-0.596775000
H	-0.318108000	-0.993748000	-2.172081000
C	-1.451868000	-4.499706000	0.296248000
C	-0.112348000	-4.627249000	0.405116000
H	-2.251645000	-5.227742000	0.398421000
H	0.512069000	-5.489503000	0.621530000
C	-0.539487000	-2.433522000	-0.090548000
N	-1.703743000	-3.170263000	0.000144000
N	0.427936000	-3.371226000	0.176742000
C	-3.041013000	-2.677726000	-0.087317000
C	-3.718882000	-2.364956000	1.100594000
C	-3.662079000	-2.572403000	-1.342423000
C	-5.024363000	-1.863157000	1.002175000
C	-4.966545000	-2.076144000	-1.389341000
C	-5.662696000	-1.704033000	-0.232966000
H	-5.561185000	-1.606305000	1.920482000

H	-5.454570000	-1.974948000	-2.363217000
C	1.834262000	-3.151393000	0.288614000
C	2.642324000	-3.296543000	-0.846590000
C	2.372410000	-2.877397000	1.555493000
C	4.021654000	-3.122457000	-0.694324000
C	3.756777000	-2.722482000	1.658962000
C	4.597845000	-2.830745000	0.545682000
H	4.664140000	-3.221517000	-1.574146000
H	4.190537000	-2.504734000	2.639497000
C	-3.083946000	-2.589543000	2.443684000
H	-2.015453000	-2.328161000	2.445322000
H	-3.596397000	-2.014295000	3.227290000
H	-3.143225000	-3.652844000	2.728811000
C	-2.929456000	-2.961373000	-2.589007000
H	-2.603824000	-4.013010000	-2.548563000
H	-3.562169000	-2.831019000	-3.477136000
H	-2.021269000	-2.345732000	-2.699860000
C	-7.070755000	-1.189946000	-0.317437000
H	-7.375039000	-0.688098000	0.611581000
H	-7.190281000	-0.481836000	-1.150780000
H	-7.777593000	-2.017362000	-0.494611000
C	1.485231000	-2.745232000	2.758922000
H	0.913523000	-3.668411000	2.944363000
H	2.074348000	-2.523152000	3.658703000
H	0.747321000	-1.938352000	2.619605000
C	2.039513000	-3.628563000	-2.179355000
H	1.240650000	-2.915380000	-2.438243000
H	2.799782000	-3.610430000	-2.971690000
H	1.582036000	-4.631344000	-2.170838000
C	6.075903000	-2.604926000	0.678299000
H	6.633915000	-3.080615000	-0.140536000
H	6.305179000	-1.526058000	0.651468000
H	6.459459000	-2.993237000	1.633222000
H	1.002854000	7.032762000	-0.542493000
C	0.538112000	6.174439000	-1.032403000
C	-0.173300000	6.363872000	-2.241824000
C	0.646281000	4.920490000	-0.474777000
C	-0.769733000	5.303159000	-2.888979000
H	-0.246991000	7.368168000	-2.665666000
C	0.041025000	3.815735000	-1.122644000
H	1.192573000	4.764268000	0.458400000
C	-0.671471000	4.004447000	-2.339357000
H	-1.319631000	5.433101000	-3.822852000
C	0.095607000	2.494880000	-0.626414000
N	-1.248665000	2.944697000	-2.967140000
H	0.610154000	2.274386000	0.312669000
N	-0.465177000	1.478241000	-1.259907000
C	-1.124260000	1.769320000	-2.430307000
H	-1.569794000	0.904027000	-2.926331000
H	6.390035000	1.898255000	2.193507000
C	5.801618000	1.572802000	1.332894000
C	6.364760000	1.647366000	0.035440000
C	4.522900000	1.094801000	1.511403000

C	5.651693000	1.243706000	-1.072660000
H	7.381010000	2.030509000	-0.083868000
C	3.771494000	0.676192000	0.386421000
H	4.078605000	1.032749000	2.507903000
C	4.335960000	0.747308000	-0.916763000
H	6.073066000	1.294204000	-2.078390000
C	2.448841000	0.185564000	0.470933000
N	3.618933000	0.343243000	-1.999047000
H	1.937653000	0.121669000	1.435020000
N	1.765790000	-0.191597000	-0.594748000
C	2.412940000	-0.101323000	-1.800079000
H	1.821428000	-0.432239000	-2.657594000
H	-0.508963000	0.066983000	0.988919000
H	-1.962354000	-0.571175000	-0.678001000
H	0.807656000	3.401618000	4.688860000
C	0.009538000	3.370208000	3.944606000
C	-0.491949000	4.577046000	3.407626000
C	-0.504907000	2.159533000	3.534321000
C	-1.500863000	4.574628000	2.464801000
H	-0.071837000	5.527089000	3.745685000
C	-1.540207000	2.135951000	2.572985000
H	-0.125870000	1.218547000	3.939372000
C	-2.047564000	3.351368000	2.027637000
H	-1.895656000	5.499763000	2.042225000
C	-2.127380000	0.934623000	2.113020000
N	-3.057197000	3.355639000	1.101956000
H	-1.912581000	-0.035441000	2.550491000
N	-3.162021000	1.028321000	1.277907000
C	-3.573640000	2.224115000	0.762506000
H	-4.391423000	2.171052000	0.038216000
H	-3.612461000	0.173491000	0.958460000

9. X Ray Data for sbd1415

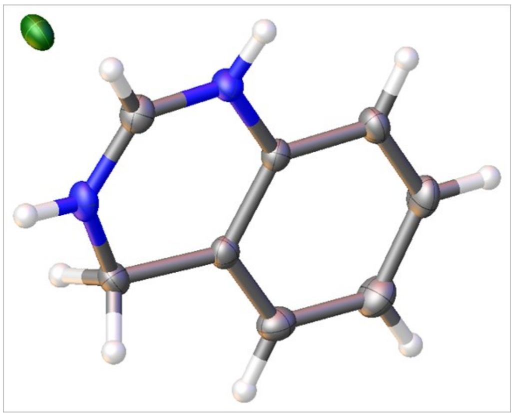

Table S23 Crystal data and structure refinement for sbd1415.

Identification code	sbd1415
Empirical formula	$C_8H_9ClN_2$
Formula weight	168.62
Temperature/K	110.00(14)
Crystal system	triclinic
Space group	P-1
a/Å	5.5421(5)
b/Å	7.9319(9)
c/Å	9.4933(3)
α/°	82.068(6)
β/°	76.113(6)
γ/°	80.977(8)
Volume/Å^3	397.91(6)
Z	2
ρ_{calc}g/cm^3	1.407
μ/mm^{-1}	0.410
F(000)	176.0
Crystal size/mm^3	0.2714 × 0.0838 × 0.0192
Radiation	MoKα (λ = 0.71073)
2Θ range for data collection/°	7.21 to 64.056
Index ranges	-7 ≤ h ≤ 7, -11 ≤ k ≤ 10, -13 ≤ l ≤ 13
Reflections collected	4030

Independent reflections	2425 [R_{int} = 0.0273, R_{sigma} = 0.0494]
Data/restraints/parameters	2425/0/108
Goodness-of-fit on F^2	1.129
Final R indexes [I>=2σ (I)]	R_1 = 0.0562, wR_2 = 0.1316
Final R indexes [all data]	R_1 = 0.0648, wR_2 = 0.1363
Largest diff. peak/hole / e Å^{-3}	0.66/-0.32

Structure solved by Natalie Pridmore

Table S24 Fractional Atomic Coordinates (×10^4) and Equivalent Isotropic Displacement Parameters (Å^2×10^3) for sbd1415. U_{eq} is defined as 1/3 of of the trace of the orthogonalised U_{IJ} tensor.

Atom	*x*	*y*	*z*	U(eq)
Cl1	8860.7(11)	2268.0(7)	3789.8(6)	20.08(15)
C1	2558(4)	2706(3)	6101(3)	20.6(4)
C2	5288(4)	3508(3)	7417(2)	16.3(4)
C3	6214(5)	4824(3)	7872(3)	20.5(4)
C4	7788(5)	4404(3)	8837(3)	24.4(5)
C5	8400(5)	2706(4)	9355(3)	26.9(5)
C6	7492(5)	1404(3)	8864(3)	23.6(5)
C7	5951(4)	1795(3)	7872(2)	17.2(4)
C8	5108(5)	385(3)	7265(3)	21.4(5)
N1	3582(4)	3925(3)	6486(2)	18.5(4)
N2	3189(4)	1089(3)	6440(2)	20.4(4)

Table S25 Anisotropic Displacement Parameters (Å^2×10^3) for sbd1415. The Anisotropic displacement factor exponent takes the form: $-2\pi^2[h^2a^{*2}U_{11}+2hka^*b^*U_{12}+\ldots]$.

Atom	U_{11}	U_{22}	U_{33}	U_{23}	U_{13}	U_{12}
Cl1	20.8(3)	14.2(2)	27.7(3)	-3.04(19)	-9.5(2)	-2.45(18)
C1	16.1(10)	24.6(12)	21.8(11)	-4.6(9)	-4.7(8)	-2.4(8)
C2	16.5(10)	17.3(10)	15.6(9)	-3.4(7)	-3.7(8)	-1.9(8)
C3	23.1(11)	17.5(10)	22.6(10)	-3.9(8)	-5.4(9)	-5.9(8)
C4	25.7(12)	28.2(13)	23.7(11)	-6.9(9)	-7.8(9)	-9.2(10)

C5	25.6(12)	33.4(14)	24.7(12)	-3.8(10)	-11.6(10)	-2.8(10)
C6	24.6(12)	24.2(12)	21.0(11)	0.2(9)	-6.9(9)	0.1(9)
C7	18.1(10)	17.5(10)	15.5(9)	-3.6(7)	-1.5(8)	-2.6(8)
C8	27.8(12)	14.8(10)	21.2(10)	-3.0(8)	-4.9(9)	-1.7(9)
N1	20.7(9)	15.5(9)	20.5(9)	-2.5(7)	-7.9(7)	-0.3(7)
N2	20.0(9)	20(1)	23.8(10)	-7.3(7)	-4.5(8)	-6.8(8)

Table S26 Bond Lengths for sbd1415.

Atom	Atom	Length/Å	Atom	Atom	Length/Å
C1	N1	1.326(3)	C4	C5	1.387(4)
C1	N2	1.293(3)	C5	C6	1.395(4)
C2	C3	1.393(3)	C6	C7	1.391(3)
C2	C7	1.388(3)	C7	C8	1.507(3)
C2	N1	1.416(3)	C8	N2	1.466(3)
C3	C4	1.385(3)			

Table S27 Bond Angles for sbd1415.

Atom	Atom	Atom	Angle/˚	Atom	Atom	Atom	Angle/˚
N2	C1	N1	123.0(2)	C7	C6	C5	120.5(2)
C3	C2	N1	119.3(2)	C2	C7	C6	118.4(2)
C7	C2	C3	121.8(2)	C2	C7	C8	121.0(2)
C7	C2	N1	118.8(2)	C6	C7	C8	120.5(2)
C4	C3	C2	118.7(2)	N2	C8	C7	110.66(19)
C3	C4	C5	120.6(2)	C1	N1	C2	120.7(2)
C4	C5	C6	119.8(2)	C1	N2	C8	124.7(2)

Table S28 Torsion Angles for sbd1415.

A	B	C	D	Angle/˚	A	B	C	D	Angle/˚
C2	C3	C4	C5	-0.9(4)	C6	C7	C8	N2	170.7(2)

C2 C7 C8 N2	-11.7(3)	C7 C2 C3 C4	-1.7(4)
C3 C2 C7 C6	3.0(3)	C7 C2 N1 C1	2.6(3)
C3 C2 C7 C8	-174.6(2)	C7 C8 N2 C1	9.1(3)
C3 C2 N1 C1	-176.1(2)	N1 C1 N2 C8	-0.8(4)
C3 C4 C5 C6	2.1(4)	N1 C2 C3 C4	177.0(2)
C4 C5 C6 C7	-0.8(4)	N1 C2 C7 C6	-175.7(2)
C5 C6 C7 C2	-1.7(4)	N1 C2 C7 C8	6.6(3)
C5 C6 C7 C8	175.9(2)	N2 C1 N1 C2	-5.8(4)

Table S29 Hydrogen Atom Coordinates (Å×10^4) and Isotropic Displacement Parameters (Å^2×10^3) for sbd1415.

Atom	*x*	*y*	*z*	U(eq)
H1	1331	3030	5563	25
H3	5784	5963	7535	25
H4	8439	5268	9140	29
H5	9413	2437	10027	32
H6	7920	265	9202	28
H8A	6534	-209	6631	26
H8B	4434	-440	8059	26
H1A	2960(60)	4970(40)	6330(30)	21(7)
H2	2480(70)	360(50)	6160(40)	43(10)

Experimental

Single crystals of $C_8H_9ClN_2$ **[sbd1415]** were obtained. A suitable crystal was selected and **analyzed** on a **SuperNova, Dual, Cu at zero, Eos** diffractometer. The crystal was kept at 110.00(14) K during data collection. Using Olex2 [19], the structure was solved with the Superflip [20] structure solution program using Charge Flipping and refined with the ShelXL [21] refinement package using Least Squares minimization.

Crystal structure determination of **[sbd1415]** Crystal Data for $C_8H_9ClN_2$ (M =168.62 g/mol): triclinic, space group P-1 (no. 2), a = 5.5421(5) Å, b = 7.9319(9) Å, c = 9.4933(3) Å, α = 82.068(6)°, β = 76.113(6)°, γ = 80.977(8)°, V = 397.91(6) Å^3, Z = 2, T = 110.00(14) K, μ(MoKα) = 0.410 mm^{-1}, $Dcalc$ = 1.407 g/cm^3, 4030 reflections measured (7.21° ≤ 2Θ ≤ 64.056°), 2425 unique (R_{int} = 0.0273, R_{sigma} = 0.0494) which were used in all calculations. The final R_1 was 0.0562 (I > 2σ(I)) and wR_2 was 0.1363 (all data).

Refinement model description

Number of restraints - 0, number of constraints - unknown.

Details:

```
1. Fixed Uiso
 At 1.2 times of:
  All C(H) groups, All C(H,H) groups
2.a Secondary CH2 refined with riding coordinates:
 C8(H8A,H8B)
2.b Aromatic/amide H refined with riding coordinates:
 C1(H1), C3(H3), C4(H4), C5(H5), C6(H6)
```

This report has been created with Olex2, compiled on 2014.09.19 svn.r3010 for OlexSys.

1. Cowley, M.J., et al., *Iridium N-Heterocyclic Carbene Complexes as Efficient Catalysts for Magnetization Transfer from para-Hydrogen.* Journal of the American Chemical Society, 2011. **133**(16): p. 6134–6137.
2. Frisch, M.J. and others, *Gaussian 09, Revision B.01*. 2010.
3. Cances, E., B. Mennucci, and J. Tomasi, *A new integral equation formalism for the polarizable continuum model: Theoretical background and applications to isotropic and anisotropic dielectrics.* Journal of Chemical Physics, 1997. **107**(8): p. 3032-3041.
4. Mennucci, B., E. Cances, and J. Tomasi, *Evaluation of solvent effects in isotropic and anisotropic dielectrics and in ionic solutions with a unified integral equation method: Theoretical bases, computational implementation, and numerical applications.* Journal of Physical Chemistry B, 1997. **101**(49): p. 10506-10517.
5. Cances, E. and B. Mennucci, *New applications of integral equations methods for solvation continuum models: ionic solutions and liquid crystals.* Journal of Mathematical Chemistry, 1998. **23**(3-4): p. 309-326.
6. Adamo, C. and V. Barone, *Toward reliable density functional methods without adjustable parameters: The PBE0 model.* Journal of Chemical Physics, 1999. **110**(13): p. 6158-6170.
7. Coquet, R., M. Tada, and Y. Iwasawa, *Energy-gaining formation and catalytic behavior of active structures in a SiO2-supported unsaturated Ru complex catalyst for alkene epoxidation by DFT calculations.* Physical Chemistry Chemical Physics, 2007. **9**(45): p. 6040-6046.
8. Zyder, M., et al., *Photochemical Reaction of Mo(CO)6 with Et2GeH2: NMR and DFT Studies of Reaction Products; Crystal Structure of a Novel Complex [{Mo(μ-η2-H−GeEt2)(CO)4}2].* Organometallics, 2009. **28**(20): p. 5857-5865.
9. Tsipis, A.C., *DFT flavor of coordination chemistry.* Coordination Chemistry Reviews, 2014. **272**: p. 1-29.
10. Weigend, F. and R. Ahlrichs, *Balanced basis sets of split valence, triple zeta valence and quadruple zeta valence quality for H to Rn: Design and assessment of accuracy.* Physical Chemistry Chemical Physics, 2005. **7**(18): p. 3297-3305.
11. Feller, D., *The role of databases in support of computational chemistry calculations.* Journal of Computational Chemistry, 1996. **17**(13): p. 1571-1586.
12. Schuchardt, K.L., et al., *Basis set exchange: A community database for computational sciences.* Journal of Chemical Information and Modeling, 2007. **47**(3): p. 1045-1052.
13. Schafer, A., H. Horn, and R. Ahlrichs, *Fully Optimized Contracted Gaussian-Basis Sets For Atoms Li To Kr.* Journal of Chemical Physics, 1992. **97**(4): p. 2571-2577.
14. Roy, L.E., P.J. Hay, and R.L. Martin, *Revised basis sets for the LANL effective core potentials.* Journal of Chemical Theory and Computation, 2008. **4**(7): p. 1029-1031.
15. Weigend, F., et al., *RI-MP2: optimized auxiliary basis sets and demonstration of efficiency.* Chemical Physics Letters, 1998. **294**(1-3): p. 143-152.
16. Grimme, S., S. Ehrlich, and L. Goerigk, *Effect of the damping function in dispersion corrected density functional theory.* Journal of Computational Chemistry, 2011. **32**(7): p. 1456-1465.
17. Boys, S.F. and F. Bernadi, *CALCULATION OF SMALL MOLECULAR INTERACTIONS BY DIFFERENCES OF SEPARATE TOTAL ENERGIES - SOME PROCEDURES WITH REDUCED ERRORS.* Molecular Physics, 1970. **19**(4): p. 553-553.

18. Simon, S., M. Duran, and J.J. Dannenberg, *How does basis set superposition error change the potential surfaces for hydrogen bonded dimers?* Journal of Chemical Physics, 1996. **105**(24): p. 11024-11031.
19. Dolomanov, O.V., Bourhis, L.J., Gildea, R.J, Howard, J.A.K. & Puschmann, H. (2009), J. Appl. Cryst. 42, 339-341.
20. Palatinus, L. & Chapuis, G. (2007). J. Appl. Cryst., 40, 786-790; Palatinus, L. & van der Lee, A. (2008). J. Appl. Cryst. 41, 975-984; Palatinus, L., Prathapa, S. J. & van Smaalen, S. (2012). J. Appl. Cryst. 45, 575-580.
21. Sheldrick, G.M. (2008). Acta Cryst. A64, 112-122.
